# Cognitively engaging sedentary activities are associated with better cognitive function in middle-aged adults: a cross-sectional study using the 1970 British Cohort Study

**DOI:** 10.1186/s44167-026-00110-5

**Published:** 2026-08-28

**Authors:** Lina Al Qadi, Daniel Saucier, Sophie Rayner, Jade Pitre, Madeleine Savoie, Josiane Stadler, Jalila Jbilou, Myles W. O’Brien

**Affiliations:** 1 Centre de Formation Médicale du Nouveau-Brunswick, 50 rue de la Francophonie, Moncton, NB E1A 7R1 Canada; 2 Department of Medicine, Faculty of Medicine and Health Sciences, Université de Sherbrooke, 2500 de L’Université Boulevard, Sherbrooke, QC J1K 2R1 Canada; 3 School of Physiotherapy, Faculty of Health, Dalhousie University, 5869 University Avenue, Halifax, NS B3H 4R2 Canada; 4 School of Psychology, Faculty of Health Sciences and Community Services, Université de Moncton, 18 Avenue Antonine-Maillet, Moncton, NB E1A 3E9 Canada

**Keywords:** Reading, Video games, Internet, Television, Verbal memory, Executive function

## Abstract

**Background:**

Prior research examining sedentary time and cognitive function has produced mixed findings with limited attention to specific types of sedentary behavior. This cross-sectional study tested the hypothesis that more cognitively active sedentary behaviors (playing video games, internet use, and reading) are positively associated with cognitive performance, in contrast to cognitively passive sedentary behavior (watching television).

**Methods:**

Data from 3,943 participants (aged 46–48; 53.3% female) with valid activPAL3 accelerometry, self-reported time spent doing sedentary behaviors, cognitive assessment scores (10-word auditory verbal learning test, category verbal fluency [animal naming] and two-letter cancellation task), and covariate data (sex assigned at birth, moderate-to-vigorous intensity physical activity [MVPA], occupational physical activity, and level of education) were cross-sectionally analyzed from the Age 46 Biomedical Sweep of the 1970 British Cohort Study. Linear regression models, including sex-stratification, evaluated associations between device-measured activity monitoring and specific sedentary behaviors with standardized cognitive assessment scores (z-scores) and a composite global cognition score (primary outcome).

**Results:**

In adjusted pooled models, MVPA was positively associated with better global cognition (β = 0.069 ± 0.021 [0.027–0.110] per hour/day, *p*_*FDR*_ = 0.009). Reading (β = 0.042 ± 0.008 [0.026–0.058] per hour/day, *p*_*FDR*_ < 0.001) and internet use (β = 0.017 ± 0.006 [0.005–0.029] per hour/day, *p*_*FDR*_ = 0.030) were positively associated with better global cognition. Watching television (β = − 0.006 ± 0.005 [− 0.017–0.004] per hour/day, *p*_*FDR*_ = 0.297) and playing video games (β = 0.015 ± 0.009 [− 0.002–0.032] per hour/day, *p*_*FDR*_ = 0.128) were not associated with global cognition.

**Conclusion:**

While the cognitively passive sedentary behavior of watching television was not associated with global cognition, the more cognitively active sedentary behaviors of reading and internet use were associated with better global cognition in middle-aged adults. These cross-sectional findings highlight potential concurrent behavioral targets for cognitive health in middle-aged adults. The data suggest that lifestyle strategies might optimize cognitive outcomes by prioritizing the accumulation of MVPA, while concurrently supporting a more cognitively active composition of unavoidable sedentary time.

**Supplementary Information:**

The online version contains supplementary material available at https://doi.org/10.1186/s44167-026-00110-5.

## Introduction

Cognitive deficits may begin to emerge in middle-age [1]. Impairments in executive functions, defined as cognitive processes enabling goal-oriented behaviors (including working memory, planning, inhibition, and cognitive flexibility) and in verbal memory (encompassing the encoding, storing, and retrieving of verbal information) are among the earliest indicators of cognitive disorders and are highly predictive of developing dementia in the future [2, 3]. Although cognitive interventions typically target older adults, middle age may represent a critical period for prevention, during which addressing modifiable risk factors could attenuate early functional decline [1]. Several modifiable risk factors have been associated with cognitive dysfunction, including poor dietary habits [4], inadequate sleep quality [5], and insufficient physical activity [6]. Pertaining to the latter, a growing body of research indicates that increased physical activity is positively associated with improvements in executive function, memory, and overall cognitive performance [7]. However, research on the impact of sedentary time on cognition has yielded equivocal results [8, 9], with some showing positive [10, 11], while others negative [12, 13] impacts on executive function, memory, and/or overall cognition. These discrepancies may be rooted in methodological differences. While a two-year follow-up study utilizing a subjective cognitive assessment tool observed negative impacts of sedentary time on cognitive abilities (age 74.6 ± 6.2 years, waist-worn accelerometer) [13], a cross-sectional study using objective cognitive assessment tests observed positive associations between sedentary time and overall cognition (age 54–56 years, waist-worn accelerometer) after adjusting for moderate-to-vigorous physical activity (MVPA) [11]. Further, a cross-sectional compositional analysis of the 1970 British Cohort Study (BCS70) using the Age 46 Sweep (thigh-worn accelerometry) observed that both MVPA and sedentary time were associated with better cognitive scores [10]. However, a common limitation among these studies is that they did not evaluate the effects of different contextual types of sedentary behavior (e.g., distinguishing between cognitively active activities such as reading and a cognitively passive activity like television viewing) on cognitive function. A more nuanced understanding of how various forms of sedentary behavior influence cognitive function is essential for advancing epidemiological research and informing targeted public health interventions aimed at promoting brain health.

Accumulating neuroimaging and epidemiological evidence strongly supports that high total volumes of sedentary behavior, particularly when prolonged and uninterrupted, are detrimental to brain health and accelerate cognitive decline through compromised cerebrovascular and metabolic pathways [8, 14, 15]. However, the notion that specific types of sedentary behavior may have neutral or even positive associations with cognitive function complicates this paradigm [8]. One possible hypothesis for this phenomenon is that sedentary time can be spent doing cognitively stimulating activities (e.g., reading as opposed to television-viewing) that may improve cognition [10, 11]. In fact, a systematic review noted that playing video games has been shown to have potential benefits for cognitive function and even result in functional brain activation changes in prefrontal, frontal, and parietal lobes [16]. However, most of the included studies focused on earlier or older adulthood, and the vast majority of current sedentary literature with cognition is focused on older adults (54 + years old) [11–13]. The behavioral topography of middle-aged adults is fundamentally distinct owing to them being still embedded in the workforce. Although a substantial portion of their daily sedentary time is likely occupational and non-discretionary, understanding how specific types of leisure sedentary behaviors affects the association between sedentary time and cognition during midlife may provide additional lifestyle intervention avenues for public health to employ beyond focusing on increasing physical activity. In fact, a cross-sectional study on adults aged 37–73 found that nonoccupational computer-use time is linked with increased cognitive function, while television time and driving were associated with decreased cognitive function [17]. However, this study solely used self-reported sedentary behaviors, with no study to our knowledge pairing such information with more robust device-based measurement tools such as accelerometers. While self-reports can offer information on the context of the sedentary time, accelerometers provide detailed information on total sedentary time, as well as allow for adjustment by physical activity such as moderate-to-vigorous intensity physical activity (MVPA) of a participant [7]. Studies have demonstrated that most adults overestimate their amount of physical activity, but also underestimate their sedentary time, highlighting the importance of device-based measurement of activity monitoring [18, 19]. Sex-stratification should also be considered as females’ activity behaviors may be more sensitive to cognitive function changes [20], as well as daily time spent in different sedentary behaviors potentially varying by sex and/or gender [21].

The objectives of the current study were to examine: (1) The association between device-based activity monitoring metrics (total sedentary time, standing, and MVPA) with global cognition (cognitive performance on a cognitive assessment module consisting of four tasks); (2) The association between specific sedentary behaviors (i.e. watching television, playing video games, on the internet, and reading) and global cognition; (3) Sex-specific associations of the above objectives. We hypothesized that MVPA would be positively associated with global cognition, while total sedentary time would not be associated with global cognition. Furthermore, we hypothesized that the different types of sedentary behaviors comprising this total sedentary time would yield divergent associations to global cognition. Specifically, that cognitively active sedentary behavior such as reading, internet browsing, and video games would show a positive association with global cognition, while passive behavior (watching television) would be negatively associated with global cognition. Finally, for sex-specific models, we hypothesized that the associations between specific sedentary behaviors and cognitive subtests would be more pronounced in females than in males and would vary based on sex-specific compositions of daily sedentary time.

## Methods

###  Study sample

 This cross-sectional study was a secondary analysis of data from Sweep 10 (age 46) of the 1970 British Cohort Study (BCS70), an ongoing longitudinal study that follows the lives of all individuals born in England, Scotland, and Wales between April 5 and April 11, 1970, with information collected on about 16,571 babies [22–24]. Sweep 10 took place between July 2016 and July 2018 (*n* = 8,581) and involved many data collection elements, including activity monitoring via a thigh-mounted accelerometer and cognitive assessments administered by nurses. Data from the device-measured activity monitoring, self-reported sedentary behaviors, and cognitive assessments were collected concurrently during the unified survey sweep, ensuring temporal alignment across all exposure metrics and outcome measures. Participants from Sweep 10 were included in the current study if they wore an accelerometer for at least 4 days with at least 10 h of valid waking wear time per day (*n* = 5,034) [25, 26]. Participants were excluded if they lacked results from any of the four tasks comprised in the cognitive assessment module (see below) or were missing information on at least one of the covariates (see below).

###  Activity monitoring

Participants were invited to wear the thigh-mounted activPAL3 micro triaxial accelerometer (PAL technologies Ltd., Glasgow, UK) continuously for up to seven days, including sleeping and bathing, in accordance with the validated wear protocol [27]. The device was waterproof, was fitted by a trained nurse on the midline anterior aspect of the upper thigh and was set to a sampling frequency of 20 Hz. The collected positional and accelerometric data were processed through the activPAL3 software and used to determine total sedentary (total sitting/lying time over the day), prolonged sedentary (total time spent sitting in bouts lasting more than 60 consecutive minutes, summed across the day), standing (total time standing, not stepping, over the day), and MVPA times (in hours per day). MVPA was defined as activity time with a cadence value of ≥ 100 steps/min, which represents a widely accepted and validated threshold for moderate-intensity activity (equivalent to ≥ 3.0 METs) in adults [28, 29]. Light intensity physical activity (LPA) was calculated as the residual from total stepping time after accounting for MVPA time, isolating lower cadence [30, 31]. Sleep time was identified using a validated algorithm as sleep may be recorded as multiple bouts of sitting or lying, interrupted by actual or mistakenly detected posture changes and steps [32]. Thus, the total waking wear time is comprised of total sedentary time + standing time + total stepping time (LPA + MVPA). While self-reported sleep (4-week recall) was available from the age 46 BCS70 questionnaire, the choice was made a priori to focus on waking habitual activity to prevent temporal asymmetry within our models (7-day device-measured waking movement).

###  Self-reported sedentary behaviors

 Leisure-time sedentary behaviors were indexed using a series of domain-specific items embedded within the BCS70 Age 46 self-completion questionnaire. This section directly adapted its overarching structural layout and response scales from the validated EPIC-Norfolk Physical Activity Questionnaire framework [33]. While the original framework included television viewing as a sedentary behavior metric, the BCS70 team modified it to comprehensively capture modern screen-based and cognitive sedentary domains (watching television, playing video games, using the internet, and reading) within the “Home Activities” subsection of their self-completion questionnaire. Time spent doing these sedentary behaviors on a typical weekday or weekend day were self-reported by participants (see Supplemental Methods 1 for wording of questions) with responses categorized as the median of the provided range: none (0 h), < 1.0 h/day (0.5 h), 1.0–1.9 h/day (1.5 h), 2.0–2.9 h/day (2.5 h), 3.0–3.9 (hours/day 3.5 h), or ≥ 4.0 h/day (5 h). The average daily time spent for each sedentary behavior was calculated as: (weekend time × 2) + (weekday time × 5) / 7. This average time was then summed across all sedentary behaviors to calculate each participant’s total self-reported sedentary time. Each behavior was then expressed as a percentage of this total self-reported sedentary time. To estimate the amount of sedentary time spent in each behavior, the respective percentage was multiplied by the participant’s activPAL-measured total sedentary time. This analysis technique is based on the premise that while the number of hours self-reported for each behaviors is likely over/under-estimated, the proportion of hours spent in each behavior are more reasonably correct [19, 34].

###  Assessment of cognitive function

 Participants were administered a cognitive assessment module consisting of four tasks: 10-Word List (adapted from Rey’s original framework [35]) Auditory Verbal Learning Test (Immediate Recall), Category Verbal Fluency Test (Animal Naming Task) [36], a Two-Letter Cancellation Task [37, 38], and 10-Word List Auditory Verbal Learning Test (Delayed Recall). First, verbal learning and memory encoding were evaluated by having participants learn one of four randomly selected lists of 10 common words [35]. After the list was read out, participants had up to two minutes to recall as many words as they could (immediate word list recall; 1 point per word recalled). Executive function and semantic retrieval were then measured by having participants name as many animals as possible within one minute (animal naming; 1 point per animal named) [39]. This was followed by a two-letter cancellation task indexing information processing speed and sustained visual attention [37, 38], in which participants crossed out target letters (‘P’ and ‘W’) as quickly and accurately as possible within one minute from a page of random letters of the alphabet. Two distinct metrics were derived from this task: a search speed score, measured by the total number of letters scanned, and an accuracy score, measured by the combined total number of target letters (‘P’ and ‘W’) that were scanned but missed. Finally, delayed retrieval was assessed by asking participants to recall the same list of 10 words from earlier (delayed word list recall; 1 point per word recalled) [35].

The two-letter cancellation accuracy score was reverse-coded so that a higher value on any of the five individual cognitive assessment test scores (10-Word Auditory Verbal Learning Test [Immediate + Delayed Recall], Category Verbal Fluency Test [Animal Naming], and Two-Letter Cancellation Task [Visual Search Speed + Accuracy]) indicates better cognitive function. These five scores were then standardized (z-scores) into a “global cognition” score computed by taking the plain average of the individual z-scores. Z-scores represent the number of standard deviations an individual’s cognitive score is from the mean score of the study sample with positive z-scores reflecting above-average cognitive function, while lower z-scores indicating the inverse. These z-scores also establish a common metric and prevent tests with larger raw numerical ranges from disproportionately weighting composite scores. This standardizing procedure ensured that each individual cognitive task contributed equal variance to the overarching global cognition score. A 10-Word Auditory Verbal Learning Test (Immediate + Delayed Recall) composite score, as well as a Two-Letter Cancellation Task [Visual Search Speed + Accuracy] composite score, were also defined and calculated by taking the plain average of the individually computed z-scores.

### Covariates

All device-measured activity monitoring regression models were adjusted for sex (assigned at birth), self-reported occupational physical activity (categorized as ‘*sitting occupation*’, ‘*standing occupation*’, ‘*physical work*’, ‘*heavy manual work*’ or ‘*not working*’), highest education qualification earned defined per the UK government as ≤ Level 2 (General Certificate of Secondary Education or below), Level 3–4, (Advanced Level Qualifications) and ≥ Level 5 (Higher Education Qualification or higher), and LPA time. Independent variables were total sedentary time, standing time, and MVPA time with the global cognition score as the primary outcome. Exploratory analyses used the 10-Word Auditory Verbal Learning Test composite score, Animal Naming, and Two-Letter Cancellation Task composite score (z-scores) as the dependent variables. These analyses were also stratified by sex (excluding sex as a covariate). Rather than running separate models, all device-measured activity metrics were entered concurrently within the same multivariable linear regression model. Thus, 12 distinct device-measured activity monitoring models (4 cognitive z-scores [global cognition + 3 subtests] x 3 groups [pooled, female, male]) were tested (see Supplementary eTables 1 and 2 for full regression parameters). Sensitivity analyses of these 12 models included evaluating the association between prolonged sedentary time and cognition (see eTable 3).

In the self-reported sedentary behavior analyses, the independent variables were represented by the estimated time spent doing specific sedentary behaviors (watching television, playing video games, on the internet, and reading) with the global cognition score as the primary outcome. The z-scores of the subtests were the dependent variables in further exploratory analyses. The covariates for these self-reported sedentary behavior models included sex, occupational physical activity, highest education qualification achieved, and MVPA time. LPA was not included as a covariate in the self-reported sedentary behavior models, as the primary aim of these models was to isolate the contextual cognitive impact of discretionary sedentary choices while controlling for higher-intensity physical activity (MVPA), rather than modelling the full 24-hour device-measured movement spectrum. These self-reported sedentary behavior analyses were also stratified by sex. Rather than running separate models, all self-reported sedentary behaviors were entered concurrently within the same multivariable linear regression model. Thus, 12 distinct self-reported sedentary behavior models (4 cognitive z-scores [global cognition + 3 subtests] x 3 groups [pooled, female, male]) were tested (see Supplementary eTables 1 and 2 for full regression parameters).

### Statistical analysis

 Characteristics of the sample, including covariates, their activity monitoring, their cognitive assessments scores and their estimated time spent doing sedentary behaviors were compared by sex. P-values were calculated using Welch Two Sample *t*-tests for continuous variables and chi-squared tests for categorical variables. Multivariate linear regression models were used to assess the association between either a participant’s device-measured activity monitoring durations (independent), or their estimated time spent doing specific sedentary behaviors (independent), and their standardized cognitive z-scores (dependent). Additional analyses included sex-stratification to explore potential differences between males and females. Prior to conducting sex-stratified analyses, cross-product interaction terms (Exposure*Sex) were introduced into the fully adjusted models. A statistically significant interaction emerged between device-measured total sedentary time and sex for both global cognition (β = − 0.028, *p* = 0.045) and the composite 10-Word Auditory Verbal Learning Test z-score (β = − 0.048, *p* = 0.049). For parsimony and to ensure a uniform presentation across all models, sex-stratified models were computed across both device-measured activities and self-reported sedentary behaviors models.

Results are presented as unstandardized coefficients (β) ± standard error, along with 95% confidence intervals and p-values (both raw and adjusted using the Benjamini-Hochberg [False Discovery Rate; *p*_*FDR*_] procedure) for linear regression models. All statistical analyses were performed in RStudio (version 2025.09.1 + 401; Posit Software, PBC) with an ⍺ level of 0.05. However, to account for multiple testing across 4 distinctive cognitive outcomes (global cognition + 3 subtests), the Benjamini-Hochberg False Discovery Rate (FDR) procedure was applied. Associations are thus discussed as statistically robust if they maintained an FDR-adjusted significance threshold of *p*_*FDR*_ < 0.05. To assess potential multicollinearity, Variance Inflation Factors (VIF) were calculated for all final regression models. The maximum observed standardized VIF was 3.07 (observed for Total Sedentary Time within the male stratum), with all other models yielding VIF values < 1.80, well below the conservative epidemiological threshold of 10 [40]. Finally, to quantify the variance explained by our regression models, we evaluated the model fit using a hierarchical block-entry linear regression approach to calculate cumulative R^2^ values. This hierarchical approach allowed us to determine both the total variance explained by the fully adjusted models (R^2^ values) and the unique variance contributed specifically by either the block of device-measured activity metrics or the block of self-reported sedentary behaviors (ΔR^2^ values).

### Patient and public involvement

Patients or the public were not involved in the design, or conduct, or reporting, or dissemination plans of our research.

## Results

Of the 8581 potential participants from the age 46 sweep, 6492 participants (75.7%) agreed to wear an accelerometer for the study (online supplemental eFig. 1). Of those, 5569 participants (85.8%) returned their monitors with useable data for at least one day of wear. Participants were excluded if they did not meet awake wear-time requirements (*n* = 535), missing MVPA data (*n* = 511), had missing self-reported sedentary behavior data (*n* = 130), had missing cognitive assessment data (*n* = 94), or had missing covariate data (*n* = 356). As such, *N* = 3943 participants (2103 female [53.3%]) aged 46 to 48 years old were included in the present study (Table 1). On average, participants wore thigh-mounted accelerometers for 15.9 ± 1.2 waking hours per day during 6.6 ± 0.9 days. Male participants had a higher height, weight, and body mass index (all *p* < 0.001), higher step count (*p* = 0.016), and more wear, total sedentary, prolonged sedentary, and LPA time than females (all *p* < 0.001; see Table 1 for group means and standard deviations). Females had a higher level of education (*p* < 0.001) and more sit to stand transitions than males (*p* < 0.001; see Table 1 for group means and standard deviations). Occupational physical activity was higher in males than in females with a higher prevalence of physical work (26.0% versus 19.3%) and heavy manual work (8.3% versus 0.7%), and a lower prevalence of having a standing occupation (9.8% versus 18.6%) (*p* < 0.001). Females had higher cognitive assessment scores for Immediate (6.83 ± 1.41 versus 6.71 ± 1.40, *p* = 0.012) and Delayed Word List Recall (5.79 ± 1.76 versus 5.55 ± 1.76, *p* < 0.001) and Letter Cancellation Speed (355.20 ± 82.33 versus 335.98 ± 81.96, *p* < 0.001) compared to males. For estimated time spent doing sedentary behaviors, females spent more time reading than males (1.23 ± 1.19 versus 0.91 ± 1.05 h/day, *p* < 0.001), while males spent more time watching television (4.81 ± 1.95 versus 4.38 ± 1.87 h/day), playing video games (0.68 ± 1.11 versus 0.50 ± 0.93 h/day), and on the internet (3.16 ± 1.67 versus 2.88 ± 1.59 h/day) than females (all *p* < 0.001).

**Table 1:** Participant characteristics, their activity monitoring, their cognitive assessments scores and their estimated time spent doing sedentary behaviors by sex

Characteristic	Pooled (*n* = 3943)	Female (*n* = 2103)	Male (*n* = 1840)	*p*-value^a^ (Female | Male)
Height (cm)	170.1 ± 9.2 [118.6–202.8]	164.0 ± 6.2 [118.6–185.6]	177.1 ± 6.8 [156.6–202.8]	< 0.001
Weight (kg)	81.3 ± 17.1 [39.8–179.2]	74.6 ± 15.9 [39.8–163.6]	89.0 ± 15.1 [49.0–179.2]	< 0.001
Body mass index (kg/m^2^)	28.1 ± 5.3 [15.2–61.2]	27.8 ± 5.9 [15.2–61.2]	28.4 ± 4.5 [17.0–55.8]	< 0.001
Race (White)^b^	3392 (95.7%)	1861 (95.9%)	1531 (95.4%)	0.530
Race (Non-White)^b^	154 (4.3%)	80 (4.1%)	74 (4.6%)	
*Highest education qualification achieved* ^*c*^				
≤ Level 2	2187 (55.5%)	1116 (53.1%)	1071 (58.2%)	< 0.001
Level 3 or 4	592 (15.0)	358 (17.0%)	234 (12.7%)	
≥ Level 5	1164 (29.5%)	629 (29.9%)	535 (29.1%)	
*Occupational physical activity*				
Sitting occupation	2007 (50.9%)	1069 (50.8%)	938 (51.0%)	< 0.001
Standing occupation	572 (14.5%)	391 (18.6%)	181 (9.8%)	
Physical work	884 (22.4%)	406 (19.3%)	478 (26.0%)	
Heavy manual work	166 (4.2%)	14 (0.7%)	152 (8.3%)	
Not working	314 (8.0%)	223 (10.6%)	91 (4.9%)	
*Device-measured activity monitoring*				
Wear days	6.6 ± 0.9 [4–8]	6.6 ± 0.8 [4–8]	6.6 ± 0.9 [4–8]	0.917
Waking wear time (hours/day)	15.9 ± 1.2 [11.6–23.6]	15.7 ± 1.2 [11.6–22.1]	16.0 ± 1.1 [12.3–23.6]	< 0.001
Total Sedentary Time (hours/day)	9.3 ± 1.9 [2.3–16.7]	9.0 ± 1.9 [2.3–16.7]	9.6 ± 1.9 [3.4–16.3]	< 0.001
Prolonged sedentary time (hours/day)	2.3 ± 1.4 [0.0–13.3]	2.2 ± 1.3 [0.0–9.1]	2.4 ± 1.4 [0.0–13.3]	< 0.001
Standing time (hours/day)	4.6 ± 1.5 [1.1–13.0]	4.7 ± 1.5 [1.1–13.0]	4.5 ± 1.5 [1.1–10.0]	< 0.001
LPA (hours/day)	1.1 ± 0.4 [0.2–3.7]	1.1 ± 0.4 [0.3–3.4]	1.2 ± 0.5 [0.2–3.7]	< 0.001
MVPA (hours/day)	0.9 ± 0.4 [0.0–3.2]	0.9 ± 0.4 [0.0–2.9]	0.8 ± 0.4 [0.0–3.2]	0.133
Step count	9479 ± 3560 [1452–27410]	9351 ± 3426 [1452–25720]	9626 ± 3702 [1546–27410]	0.016
Sit to stand transitions	55.3 ± 15.7 [9–133]	56.8 ± 15.1 [9–133]	53.5 ± 16.2 [18–122]	< 0.001
*Cognitive assessments*				
Immediate word list Recall score	6.77 ± 1.40 [1–10]	6.83 ± 1.41 [1–10]	6.71 ± 1.40 [2–10]	0.012
Delayed word list Recall score	5.68 ± 1.77 [0–10]	5.79 ± 1.76 [0–10]	5.55 ± 1.76 [0–10]	< 0.001
Animal naming score	24.02 ± 6.06 [1–70]	24.10 ± 5.94 [1–57]	23.94 ± 6.20 [3–70]	0.39
Two-letter cancellation Accuracy Score^e^	− 3.98 ± 3.70 [− 50–0]	− 4.06 ± 3.84 [-50–0]	− 3.89 ± 3.53 [− 38–0]	0.15
Two-letter cancellation Speed score	346.23 ± 82.71 [59–780]	355.20 ± 82.33 [128–770]	335.98 ± 81.96 [59–780]	< 0.001
*Self-reported sedentary behaviors* ^*f*^				
Watching TV (estimated hours/day)	4.58 ± 1.92 [0.00–15.00]	4.38 ± 1.87 [0.00–11.82]	4.81 ± 1.95 [0.00–15.00]	< 0.001
Playing video games (estimated hours/day)	0.58 ± 1.02 [0.00–6.72]	0.50 ± 0.93 [0.00–6.72]	0.68 ± 1.11 [0.00–6.14]	< 0.001
On the internet (estimated hours/day)	3.01 ± 1.63 [0.00–13.32]	2.88 ± 1.59 [0.00–13.32]	3.16 ± 1.67 [0.00–10.74]	< 0.001
Reading (estimated hours/day)	1.08 ± 1.14 [0.00–8.56]	1.23 ± 1.19 [0.00–8.56]	0.91 ± 1.05 [0.00–6.92]	< 0.001

Data presented as mean ± SD [range] or n (%). Abbreviations: LPA = Light Intensity Physical Activity; MVPA = Moderate-to-Vigorous Physical Activity

^a^*p*-values were calculated using Welch Two Sample t-tests for continuous variables and chi-squared tests for categorical variables

^b^Information on race was missing from 397 participants in the study sample

^c^Extracted from Sweep 9 (age 42) of the 1970 British Cohort Study. ≤ Level 2 = General Certificate of Secondary Education or below; Level 3 or 4 = Advanced Level Qualifications; ≥ Level 5 = Higher Education Qualification or higher

^d^Waking wear time is comprised of Total Sedentary Time + Standing Time + Total Stepping Time (LPA + MVPA)

^e^Letter Cancellation Accuracy Score (number of misses) was reverse-coded (negative) so that a higher score (best = 0) indicates better task performance

^f^Time spent doing sedentary behaviors is estimated by expressing each behavior as a percentage of a participant’s total self-reported sedentary time multiplied by their activPAL-measured sedentary time

Using hierarchical regression, we evaluated the independent contribution of the device-measured activities block (total sedentary time, standing time, and MVPA) and the self-reported sedentary behaviors block (watching television, playing video games, internet use, and reading) on the variance explained within models. For the primary outcome of global cognition, the addition of device-measured activities (Baseline R^2^ = 0.135, Block ΔR^2^ = 0.003, *p* = 0.005, Table 2) and the addition of self-reported sedentary behaviors (Baseline R^2^ = 0.137, Block ΔR^2^ = 0.011, *p* < 0.001) yielded statistically significant increases in explained variance in comparison to a baseline model (covariates only). In sex-stratified models, the device measured activities block increased the variance explained for global cognition in males (Baseline R^2^ = 0.140, Block ΔR^2^ = 0.144, *p* = 0.034, Table 3), but not in females (Baseline R^2^ = 0.133, Block ΔR^2^ = 0.135, *p* = 0.274). For self-reported sedentary behaviors, the addition of the self-reported sedentary behaviors block increased the explained variance for global cognition in both males (Baseline R^2^ = 0.141, Block ΔR^2^ = 0.013, *p* < 0.001) and females (Baseline R^2^ = 0.135, Block ΔR^2^ = 0.009, *p* < 0.001). For increases in variance explained for specific cognitive subtest models, consult Table 2 (pooled sample) and Table 3 (sex-stratified). Full regression parameters of all 24 distinct models can also be consulted in Supplementary eTable 1 (pooled) and Supplementary eTable 2 (sex-stratified).

**Table 2:** Hierarchical regression model fit and variance explained for pooled sample

Cognitive outcome model	Model predictor block	Baseline *R*^2^	Total *R*^2^	Block Δ*R*^2^	F-test sig. (*p*-value)
Global cognition	Device-measured activities	0.135	0.138	0.003	0.005
	Self-reported sedentary Behaviors	0.137	0.148	0.011	< 0.001
10-word auditory verbal learning test (Immediate + Delayed recall)	Device-measured activities	0.095	0.096	0.002	0.082
	Self-reported sedentary Behaviors	0.095	0.103	0.008	< 0.001
Category verbal fluency test (Animal naming)	Device-measured activities	0.075	0.076	0.001	0.217
	Self-reported sedentary behaviors	0.075	0.086	0.010	< 0.001
Two-letter cancellation task (Visual search speed + Accuracy)	Device-measured activities	0.047	0.050	0.002	0.020
	Self-reported sedentary behaviors	0.048	0.050	0.002	0.089

Device-measured activities block includes: Total sedentary time, standing time, and MVPA (adjusted for sex, occupational physical activity, level of education, and LPA). Self-report sedentary behaviors block includes: Watching TV, Playing Video Games, On the Internet, and Reading (adjusted for sex, occupational physical activity, level of education, and MVPA). Models were constructed sequentially: Covariates established the baseline R^2^; Fully adjusted models indicate Total *R*^2^; Block Δ*R*^2^ represents the unique variance contributed by either the Device-Measured Activity Block or the Self-Reported Sedentary Behaviors block. LPA = Light Physical Activity; MVPA = Moderate-to-Vigourous Physical Activity

**Table 3:** Hierarchical regression model fit and variance explained stratified by sex

Cognitive outcome model	Stratum	Model predictor block	Baseline *R*^2^	Total *R*^2^	Block Δ*R*^2^	F-test sig. (*p*-value)
Global cognition	Female	Device-measured activities	0.133	0.135	0.002	0.274
		Self-reported sedentary behaviors	0.135	0.144	0.009	< 0.001
	Male	Device-Measured Activities	0.140	0.144	0.004	0.034
		Self-reported sedentary behaviors	0.141	0.154	0.013	< 0.001
10-word auditory verbal learning test (Immediate + Delayed recall)	Female	Device-measured activities	0.086	0.087	0.001	0.589
		Self-reported sedentary behaviors	0.087	0.095	0.008	< 0.001
	Male	Device-measured activities	0.104	0.107	0.003	0.072
		Self-reported sedentary behaviors	0.104	0.114	0.009	< 0.001
Category verbal fluency test (Animal naming)	Female	Device-measured activities	0.097	0.098	0.001	0.453
		Self-reported sedentary behaviors	0.097	0.107	0.011	< 0.001
	Male	Device-measured activities	0.068	0.070	0.001	0.551
		Self-reported sedentary behaviors	0.069	0.079	0.010	< 0.001
Two-letter cancellation task (Visual search speed + Accuracy)	Female	Device-measured activities	0.035	0.038	0.003	0.070
		Self-reported sedentary behaviors	0.037	0.039	0.002	0.318
	Male	Device-measured activities	0.045	0.048	0.003	0.182
		Self-reported sedentary behaviors	0.045	0.048	0.004	0.132

Device-measured activities block includes: Total sedentary time, standing time, and MVPA (adjusted for occupational physical activity, level of education, and LPA). Self-reported sedentary behaviors block includes: Watching TV, Playing Video Games, On the Internet, and Reading (adjusted for occupational physical activity, level of education, and MVPA). Models were constructed sequentially: Covariates established the baseline R^2^; Fully adjusted models indicate Total R^2^; Block ΔR^2^ represents the unique variance contributed by either the Device-Measured Activity Block or the Self-Reported Sedentary Behaviors block. LPA = Light Physical Activity; MVPA = Moderate-to-Vigourous Physical Activity

**Fig. 1:**
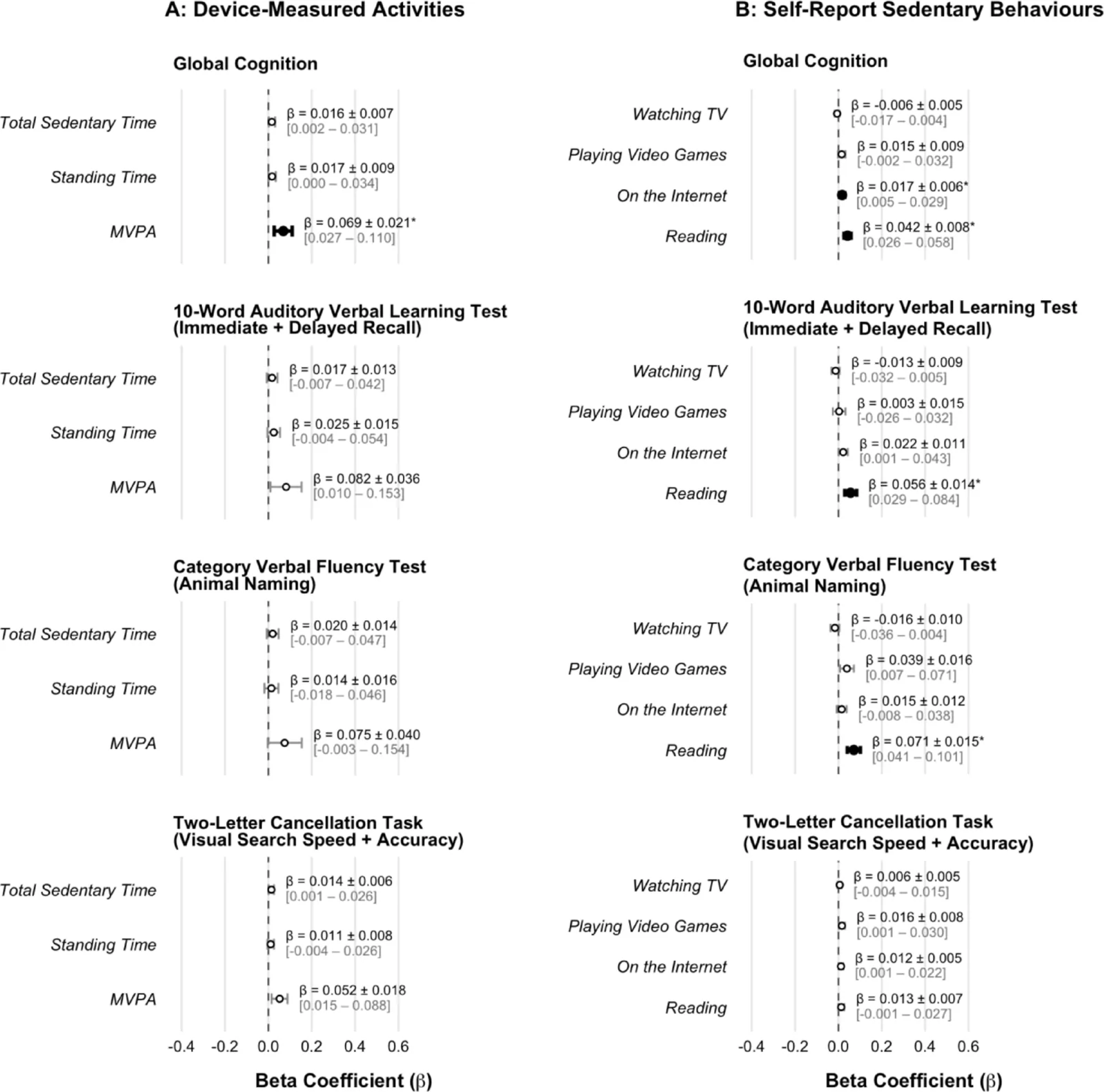
Forest plot of multivariate linear regression models for the relation between **A** device-measured activity monitoring time, **B** self-report time spent doing sedentary behaviors, and cognition (global cognition and individual subtests) with adjustment for sex, occupational physical activity and level of education. Device-measured activity monitoring models also adjust for LPA time, while self-report sedentary behaviors models also adjust for MVPA time. Results are presented as unstandardized beta coefficients with 95% confidence intervals per 1-hour increment for a given activity monitoring time or sedentary behavior. Data labels indicate β ± standard error [95% confidence interval]. Asterisks indicate associations that remained statistically significant after Benjamini-Hochberg False Discovery Rate correction (*p*_*FDR*_<0.05). Full regression parameters for all covariates are available for transparency in Supplemental Materials eTable 1. LPA = Light Physical Activity; MVPA = Moderate-to-Vigorous Physical Activity

After adjusting for multiple comparisons using the Benjamini-Hochberg FDR, MVPA was positively associated with the primary outcome of global cognition (β = 0.069 ± 0.021 per hour/day, *p* = 0.001, *p*_*FDR*_ = 0.009, Fig. 1A). In contrast, the associations between device-measured total sedentary time (β = 0.016 ± 0.007 per hour/day, *p* = 0.027) and standing time (β = 0.017 ± 0.009 per hour/day, *p* = 0.048) with global cognition did not survive multiple comparison adjustments (*p*_*FDR*_= 0.070 and *p*_*FDR*_= 0.087, respectively). For exploratory analyses, none of the device-measured activity metrics survived multiple comparison adjustments (all *p*_*FDR*_> 0.084). For self-reported sedentary behavior models, both reading (β = 0.042 ± 0.008 per hour/day, *p* < 0.001, *p*_*FDR*_< 0.001, Fig. 1B) and internet use (β = 0.017 ± 0.006 per hour/day, *p* = 0.007, *p*_*FDR*_ = 0.030) were positively associated with global cognition. Watching television (β = − 0.006 ± 0.005 per hour/day, *p* = 0.241, *p*_*FDR*_ = 0.297) and playing video games (β = 0.015 ± 0.009 per hour/day, *p* = 0.079, *p*_*FDR*_ = 0.128) were not associated with global cognition. After adjusting for multiple comparisons, significant associations between sedentary behaviors and cognitive subtests remained only for reading. Reading was positively associated with better performances on the 10-Word Auditory Verbal Learning Test (β = 0.056 ± 0.014 per hour/day, *p* < 0.001, *p*_*FDR*_ = 0.001) and Animal Naming (β = 0.071 ± 0.015 per hour/day, *p* < 0.001, *p*_*FDR*_< 0.001). In a sensitivity analysis, prolonged sedentary time was not associated with global cognition or individual cognitive subtests (all *p*_*FDR*_> 0.146, Supplementary eTable 3). Unadjusted activity monitoring and sedentary behavior models can be consulted in Supplemental eTable 4.

**Fig. 2:**
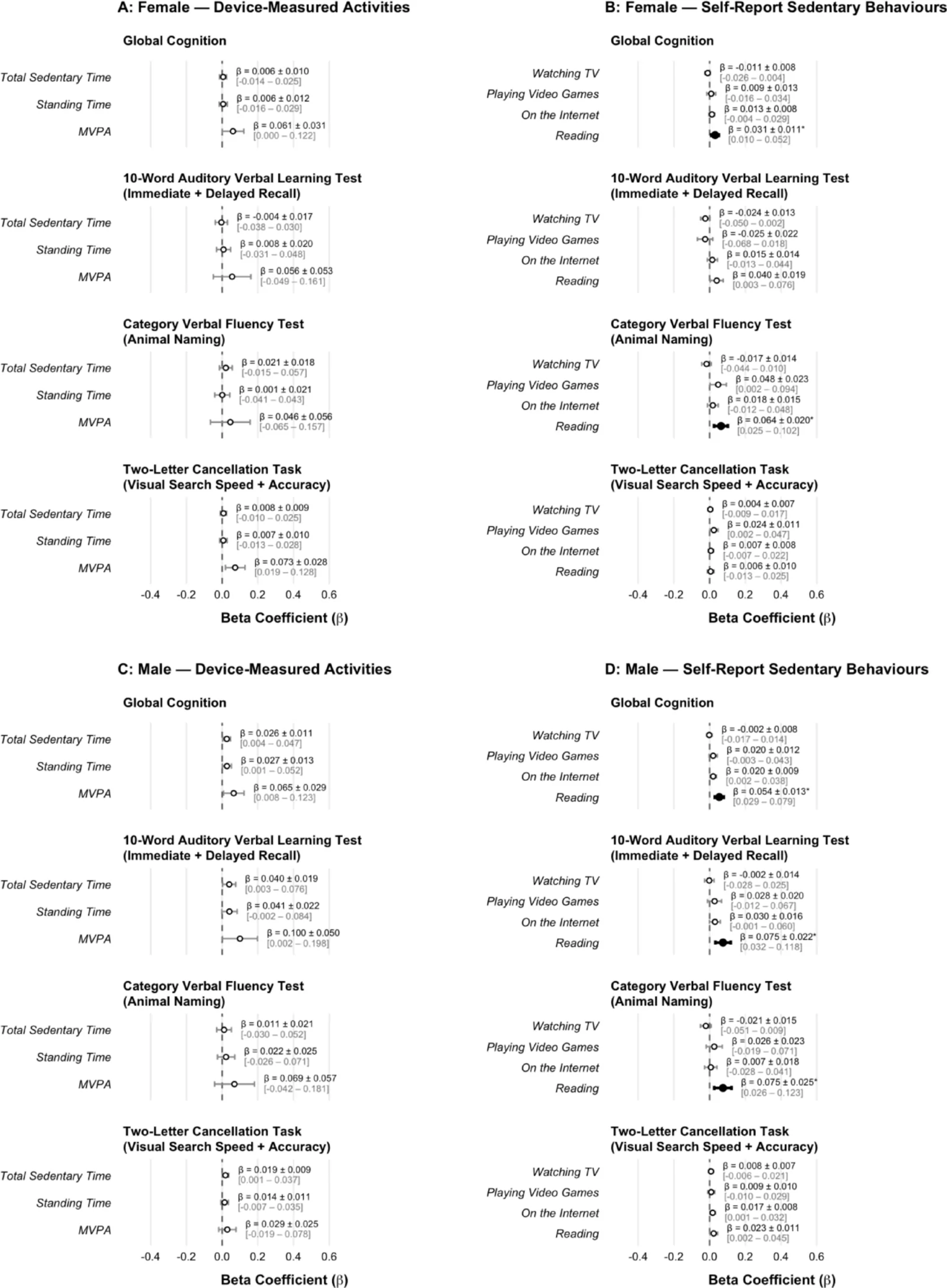
Forest plot of multivariate linear regression models for the relation between (**A**,** C**) device-measured activity monitoring time, (**B**,** D**) self-report time spent doing sedentary behaviors, and cognition (global cognition and individual subtests) stratified by sex (Top panels = Female, Bottom panels = Male). All models are adjusted for occupational physical activity and level of education. Device-mesured activity monitoring models also adjust for LPA time, while self-report sedentary behaviors models also adjust for MVPA time. Results are presented as unstandardized beta coefficients with 95% confidence intervals per 1-hour increment for a given activity monitoring time or sedentary behavior. Data labels indicate β ± standard error [95% confidence interval]. Asterisks indicate associations that remained statistically significant after Benjamini-Hochberg False Discovery Rate correction (*p*_*FDR*_<0.05). Full regression parameters for all covariates are available for transparency in Supplemental Materials eTable 2. LPA = Light Physical Activity; MVPA = Moderate-to-Vigorous Physical Activity

In females, after adjusting for multiple comparisons, there were no associations between device-measured activity metrics and global cognition (all *p*_*FDR*_>0.087, Fig. 2A). Specifically, MVPA shifted to a non-significant association with global cognition (β = 0.061 ± 0.031 per hour/day, *p* = 0.0496, *p*_*FDR*_ = 0.087) and with Two-Letter Cancellation Task performance (β = 0.073 ± 0.028 per hour/day, *p* = 0.008, *p*_*FDR*_ = 0.084). Total sedentary time (β = 0.006 ± 0.010 per hour/day, *p* = 0.560, *p*_*FDR*_ = 0.609) and standing time (β = 0.006 ± 0.012 per hour/day, *p* = 0.580, *p*_*FDR*_ = 0.609) were not associated with global cognition in females. For self-reported sedentary behaviors, reading was positively associated with global cognition in females (β = 0.031 ± 0.011 per hour/day, *p* = 0.004, *p*_*FDR*_= 0.020, Fig. 2B). Watching television (β = − 0.011 ± 0.008 per hour/day, *p* = 0.134, *p*_*FDR*_=0.176), playing video games (β = 0.009 ± 0.013 per hour/day, *p* = 0.463, *p*_*FDR*_ = 0.540), and internet use (β = 0.013 ± 0.008 per hour/day, *p* = 0.127, *p*_*FDR*_ = 0.176) were not associated with global cognition in females. For exploratory analyses, after adjusting for multiple comparisons, reading was positively associated with better Animal Naming performance in females (β = 0.064 ± 0.020 per hour/day, *p* = 0.001, *p*_*FDR*_=0.012). In a sensitivity analysis, prolonged sedentary time was not associated with global cognition or individual cognitive subtests in females (all *p*_*FDR*_>0.551, Supplementary eTable 3). Unadjusted activity monitoring and sedentary behavior models stratified by sex can be consulted in Supplemental eTable 5.

In males, after adjusting for multiple comparisons, there was no associations between device measured activity metrics and global cognition (all *p*_*FDR*_> 0.062, Fig. 2C). Total Sedentary Time (β = 0.026 ± 0.011 per hour/day, *p* = 0.018, *p*_*FDR*_=0.062), Standing Time (β = 0.027 ± 0.013 per hour/day, *p* = 0.039, *p*_*FDR*_ = 0.081), and MVPA (β = 0.065 ± 0.029 per hour/day, *p* = 0.026, *p*_*FDR*_ = 0.070) all shifted to a non-significant association with global cognition. For self-reported sedentary behaviors, reading was positively associated with global cognition in males (β = 0.054 ± 0.013 per hour/day, *p* < 0.001, *p*_*FDR*_<0.001, Fig. 2D). However, internet use shifted to a non-significant association with global cognition in males after adjustment for multiple comparisons (β = 0.020 ± 0.009 per hour/day, *p* = 0.031, *p*_*FDR*_ =0.072). Watching television (β = − 0.002 ± 0.008 per hour/day, *p* = 0.827, *p*_*FDR*_=0.827) and playing video games (β = 0.020 ± 0.012 per hour/day, *p* = 0.087, *p*_*FDR*_ = 0.131) were not associated with global cognition. For exploratory analyses, after adjusting for multiple comparisons, reading was positively associated with better performances on the 10-Word Auditory Verbal Learning Test (β = 0.075 ± 0.022 per hour/day, *p* < 0.001, *p*_*FDR*_ =0.006) and Animal Naming (β = 0.075 ± 0.025 per hour/day, *p* = 0.002, *p*_*FDR*_ = 0.018). In a sensitivity analysis, prolonged sedentary time was not associated with global cognition or individual cognitive subtests in males (all *p*_*FDR*_>0.110, Supplementary eTable 3) Unadjusted activity monitoring and sedentary behavior models stratified by sex can be consulted in Supplemental eTable 5.

## Discussion

The purpose of this cross-sectional study was to evaluate the association between device-measured total sedentary time with cognitive function in middle-aged adults, as well as the association between specific self-reported sedentary behaviors (i.e., television watching, playing video games, on the internet, and reading) and cognitive function in middle-aged adults. While no association was observed between total sedentary time and cognitive function after adjustment for multiple comparisons, MVPA was positively associated with better global cognition in the pooled sample. In contrast to our hypothesis, watching television was not associated with global cognition. In alignment with our hypothesis, the more cognitively active sedentary behaviors of reading and time spent on the internet were positively associated with global cognition in the pooled sample even after adjustment for multiple comparisons. In sex-stratified models, results were largely similar between sexes, contrary to our initial hypotheses regarding sex-specific differences, with only reading being consistently and positively associated with global cognition in both females and males after adjustment for multiple comparisons. Collectively, this suggests that cognitively active sedentary activities, such as internet use and particularly reading, are positively associated with cognitive function, supporting that the context by which total sedentary time is accumulated may influence its relation to cognitive health among middle-aged persons.

Among the sedentary behaviors examined, reading emerged as the most consistently associated with better global cognition. It was positively associated with global cognition and better performance on the Category Verbal Fluency Test (Animal Naming) in both the pooled sample and sex-stratified analyses, as well as better performance on the 10-Word Auditory Verbal Learning Test in the pooled sample and in males. The underlying cognitive processes of these subtests have been shown to be important in reading processes with executive function aiding by integrating decoded words into mental models and switching between reading and comprehension monitoring [41]. Further, using a scale of leisure activity to measure baseline reading activity and the Short Portable Mental Status Questionnaire to measure cognitive performance, a longitudinal study observed that reading was protective of cognitive function later in life [42]. While passive sedentary behavior such as television viewing did not demonstrate a significant negative association with cognition in the current study, the robust positive associations observed for reading suggest that the associations between sedentary time and cognition is context-dependent. Importantly, the lack of a significant cross-sectional association here does not imply television viewing is a benign behavior, as it may present a negative trend over a longer latency period or carry established risks for cardiometabolic and physical health metrics not captured in this analysis [43, 44]. Although more studies are needed to support the current cross-sectional findings, public health strategies could focus on cognitive enrichment opportunities within sedentary bouts rather than viewing all sedentary time as a uniform risk factor. Encouraging individuals to reallocate portions of sedentary leisure time from cognitively passive television watching to more engaging habits like reading may serve as an accessible cognitive enrichment opportunity. Thus, this approach may be particularly valuable for individuals facing significant barriers to physical activity, such as a lack of time, space, motivation, or underlying health conditions, which make increasing movement more challenging [45–47].

The current findings observed that MVPA was positively associated with global cognition and relatively more so than the investigated sedentary behaviors, including reading. Broader evidence from the literature supports that exercise promotes cognitive health [7]. Accordingly, public health messaging should continue to prioritize the accumulation of physical activity as the primary behavioral target for cognitive health. While causal pathways cannot be inferred from our cross-sectional analysis, these findings suggest that sedentary bouts may warrant further investigation as a potential window for cognitive enrichment. These data highlight that from a cognitive perspective, the qualitative composition of sedentary time may matter more than the total volume of sitting alone. While the relations between sedentary behavior, physical activity and cognition may be complex, the combination of physical activity and cognitive games (e.g. Sudoku, computer games, board games) in close temporal proximity has been associated with better cognitive performance and gray matter volume in older adults [48]. This may be supported by acute neurophysiological mechanisms. Functional neuroimaging during cognitively active sedentary behaviors like reading demonstrates robust neural activation across a distributed network, consistently involving the prefrontal cortex and parietal-temporal regions associated with language integration, comprehension, and executive function [49, 50]. Within the midlife literature, a cross-sectional study on middle-aged adults observed that a high variety of cognitively stimulating leisure activities is related to better cognitive performance [51]. Further, physical activity in midlife has been observed to be associated with later-life memory change [52]. Equity-focused interventions, such as increasing accessibility to libraries, affordable digital literacy programs, and adequate spaces for physical activity, are thus all potential avenues for reducing barriers to acting living and cognitive engagement [47, 53], thereby maximizing cognitive health benefits for all.

The divergence of our findings from prior epidemiological literature regarding total sedentary time and television viewing not displaying negative associations with global cognition may be attributable to methodological advancements and the middle-aged study sample. Previous studies showing these negative associations did not consider device-measured physical activity [17, 54]. By leveraging thigh-worn activPAL accelerometers to include MVPA as a covariate, our models successfully isolated the independent associations of sedentary behaviors. While the rationale for this is unclear, middle-aged adults may accumulate sufficient high-intensity physical activity to buffer potential risks, or they may engage in higher-quality sedentary behaviors that independently support cognitive health at this life stage. Supporting this, Mitchell et al. (2023) observed in a cross-sectional compositional analysis of the BCS70 Age 46 Sweep that both MVPA and sedentary time were positively associated with higher cognitive scores [10], reflecting that sitting time in midlife is not universally detrimental. Furthermore, crude television duration metrics fail to capture critical behavioral nuances, such as modern “binge-watching” patterns versus highly selective, cognitively engaging viewing profiles (e.g., complex narratives or educational media) that may modify cognitive impact. Additionally, the narrow middle-aged life stage (ages 46–48) represented in the BCS70 may represent a time window where structural brain changes, such as microvascular disease or advanced white matter hyperintensities, are not yet relatively widespread [55, 56]. The systemic metabolic detriments of prolonged passive sitting may also require a longer latency period or older biological age to manifest as accelerated neurodegeneration. As opposed to our cross-sectional study, a recent cohort study (aged 35–64 years, median follow-up of 19.2 years, physical activity questionnaire) noted that mentally passive sedentary behavior (e.g. watching television) was positively associated with increased risk of dementia [43]. Future longitudinal studies that extend into older adulthood while also considering device-measured physical activity and detailed media consumption characteristics are needed to discern whether television viewing in midlife truly presents as cognitively neutral and if activities like reading or internet use are independently bolstering cognitive reserve before clinical cognitive decline begins.

This study’s strengths include a large nationally representative birth cohort, device-based measurement of total and prolonged sedentary time with thigh-worn activPAL accelerometers, and standardized objective measurements for cognition. All participants being aged 46 to 48 also negates the confounding aspect of age. Combining device measured total sedentary time with self-reported sedentary behaviors measures for the estimation of time spent in these behaviors also allowed for a certain level of adjustment for potential recall bias. However, there are still some limitations with a predominantly white United Kingdom population potentially limiting generalizability to other cultural and socioeconomic contexts in which social and physical environment factors may influence sedentary behaviors such as television time [57]. Also, while potential underestimation of self-reported sedentary time in specific sedentary behaviors was adjusted by using device-measured total sedentary time, not all sedentary behaviors are necessarily covered by the four investigated in the current study. Thus, there may be other unaccounted for sedentary behaviors, such as solving a puzzle or talking while sitting, contributing to the total sedentary time. While mapping self-reported behavioral proportions onto device-measured activPAL total sedentary time minimizes absolute recall bias, it assumes that participants’ relative allocation of time across behaviors is perfectly accurate. Furthermore, the self-report questionnaire treated these sedentary behaviors as mutually exclusive, non-overlapping categories. In reality, modern sedentary habits can involve concurrent behaviors (e.g., browsing the internet on a smartphone while watching television), which can lead to misallocation or underestimation [58]. To mitigate these inaccuracies, future studies should consider using diaries as they can capture overlapping concurrent behaviors. Deploying real-time ecological momentary assessment is also possible [59], albeit introduces potential participant reactivity and monitoring burden. This study also strictly focused on a waking behavioral matrix and did not utilize a composition data analysis inclusive of sleep. Finally, this study implements a cross-sectional design and therefore is unable to exclude reverse causality (i.e. people with better cognitive function are more likely to engage in cognitively-active sedentary behaviors) or evaluate long-term effects.

## Conclusions

Overall, this study documents the relation between total sedentary time and specific sedentary behaviors with cognition. These data suggest that the association of total sedentary time on cognition depends on what people do while sedentary rather than total sedentary time alone. However, our findings also observed that MVPA maintains the most pronounced positive association with global cognition. Consequently, these findings highlight MVPA and cognitively engaging sedentary activities (such as reading) as potential concurrent targets for future lifestyle interventions aimed at preserving cognitive health.

## Supplementary Information

Below is the link to the electronic supplementary material.

Supplementary Material 1

## Data Availability

The datasets supporting this study are available in the UK Data Service repository (1970 British Cohort Study): [https://beta.ukdataservice.ac.uk/datacatalogue/series/series? id=200001]. Data are available to researchers who register with the UK Data Service and agree to the End User License Agreement.

## Abbreviations

LPA: Light physical activity
MVPA: Moderate-to-vigourous physical activity
