# Supplementary material for: Cognitively engaging sedentary activities are associated with better cognitive function in middle-aged adults: a cross-sectional study using the 1970 British Cohort Study
Source: J Act Sedentary Sleep Behav. 2026 Aug 28;5:19. doi: 10.1186/s44167-026-00110-5 (PMC13670469; doi:10.1186/s44167-026-00110-5)
Supplement: Supplementary file 1 [file 44167_2026_110_MOESM1_ESM.docx]

**Supplementary Material Contents**

**Supplemental Methods 1.** Self-reported sedentary behaviors questions from 1970 British Cohort Study: 2016-18 Survey (Self-completion Questionnaire).

**Supplemental eFigure 1.** Flow diagram indicating derivation of analytical sample (n = 3943).

**Supplemental eTable 1.** Full multivariate linear regression parameters of all 8 distinct device-measured activity monitoring and self-reported sedentary behavior models.

**Supplemental eTable 2.** Full multivariate linear regression parameters of all 16 distinct device-measured activity monitoring and self-reported sedentary behavior sex-stratified models.

**Supplemental eTable 3.** Prolonged sedentary time device-measured activity monitoring sensitivity analysis models (pooled and sex-stratified).

**Supplemental eTable 4.** Unadjusted device-measured activities models.

**Supplemental eTable 5.** Unadjusted self-reported sedentary behavior models.

**Supplemental Methods 1.** Self-reported sedentary behaviors questions from 1970 British Cohort Study: 2016-18 Survey (Self-completion Questionnaire).

Watching television

How many hours do you spend watching television programmes, videos, DVDs or Blu-ray?

Please include time spent watching programmes or films on a computer.

Playing video games

How many hours do you spend playing electronic games on a computer or games system, such as Wii, Nintendo D-S, X-Box or PlayStation?

Please also include time spent playing games on smartphones or tablets.

On the internet

How many hours do you spend using the internet at home?

Include time spent using the internet on tablets, smartphones and other mobile devices as well as computers and laptops.

Please include time spent on social networking sites such as Facebook or Twitter.

Reading

How many hours do you spend reading books (including in electronic format, e.g. Kindle) at home?

For each of the above questions, respondents used the below scale to indicate hours spent doing the respective sedentary behavior:

**Supplemental eFigure 1.** Flow diagram indicating derivation of analytical sample (n = 3943). MVPA = Moderate-to-Vigorous Physical Activity.

**Supplemental eTable 1.** Full multivariate linear regression parameters of all 8 distinct device-measured activity monitoring and self-reported sedentary behavior models.

| **Model** | **Term** | **Beta** | **SE** | **Lower 95%CI** | **Upper 95%CI** | ***p*-value** | ***p_FDR_*** |
| --- | --- | --- | --- | --- | --- | --- | --- |
| **Outcome: Global Cognition**  **Exposure: Device-Measured Activities (per hour)** | (Intercept) | -0.293 | 0.113 | -0.515 | -0.071 | 0.010 | NA |
|  | Total Sedentary Time | 0.016 | 0.007 | 0.002 | 0.031 | 0.027 | 0.070 |
|  | Standing Time | 0.017 | 0.009 | 0.000 | 0.034 | 0.048 | 0.087 |
|  | LPA | -0.020 | 0.023 | -0.065 | 0.026 | 0.402 | NA |
|  | MVPA | 0.069 | 0.021 | 0.027 | 0.110 | 0.001 | 0.009 |
|  | Female (Ref=Male) | 0.070 | 0.017 | 0.036 | 0.103 | 0.000 | NA |
|  | Education Level 3 or 4  (Ref=≤Level 2) | 0.147 | 0.024 | 0.101 | 0.194 | 0.000 | NA |
|  | Education ≥ Level 5  (Ref=≤Level 2) | 0.340 | 0.019 | 0.302 | 0.378 | 0.000 | NA |
|  | Standing Occupation (Ref=Sitting Occupation) | -0.018 | 0.025 | -0.067 | 0.031 | 0.475 | NA |
|  | Physical Work  (Ref=Sitting Occupation) | -0.137 | 0.023 | -0.183 | -0.092 | 0.000 | NA |
|  | Heavy Manual Work  (Ref=Sitting Occupation) | -0.180 | 0.044 | -0.266 | -0.094 | 0.000 | NA |
|  | Not Working  (Ref=Sitting Occupation) | -0.240 | 0.031 | -0.301 | -0.179 | 0.000 | NA |
| **Outcome: 10-Word Auditory Verbal Learning Test (Immediate + Delayed Recall)**  **Exposure: Device-Measured Activities (per hour)** | (Intercept) | -0.301 | 0.194 | -0.681 | 0.080 | 0.122 | NA |
|  | Total Sedentary Time | 0.017 | 0.013 | -0.007 | 0.042 | 0.170 | 0.255 |
|  | Standing Time | 0.025 | 0.015 | -0.004 | 0.054 | 0.092 | 0.176 |
|  | LPA | -0.039 | 0.040 | -0.117 | 0.039 | 0.331 | NA |
|  | MVPA | 0.082 | 0.036 | 0.010 | 0.153 | 0.025 | 0.127 |
|  | Female (Ref=Male) | 0.084 | 0.029 | 0.027 | 0.142 | 0.004 | NA |
|  | Education Level 3 or 4  (Ref=≤Level 2) | 0.181 | 0.041 | 0.102 | 0.261 | 0.000 | NA |
|  | Education ≥ Level 5  (Ref=≤Level 2) | 0.481 | 0.033 | 0.416 | 0.546 | 0.000 | NA |
|  | Standing Occupation (Ref=Sitting Occupation) | -0.091 | 0.043 | -0.176 | -0.007 | 0.035 | NA |
|  | Physical Work  (Ref=Sitting Occupation) | -0.229 | 0.040 | -0.307 | -0.152 | 0.000 | NA |
|  | Heavy Manual Work  (Ref=Sitting Occupation) | -0.241 | 0.075 | -0.389 | -0.093 | 0.001 | NA |
|  | Not Working  (Ref=Sitting Occupation) | -0.304 | 0.054 | -0.409 | -0.198 | 0.000 | NA |
| **Outcome: Category Verbal Fluency Test (Animal Naming)**  **Exposure: Device-Measured Activities (per hour)** | (Intercept) | -0.415 | 0.213 | -0.832 | 0.002 | 0.051 | NA |
|  | Total Sedentary Time | 0.020 | 0.014 | -0.007 | 0.047 | 0.156 | 0.352 |
|  | Standing Time | 0.014 | 0.016 | -0.018 | 0.046 | 0.395 | 0.484 |
|  | LPA | 0.027 | 0.044 | -0.058 | 0.113 | 0.533 | NA |
|  | MVPA | 0.075 | 0.040 | -0.003 | 0.154 | 0.059 | 0.206 |
|  | Female (Ref=Male) | -0.004 | 0.032 | -0.066 | 0.059 | 0.911 | NA |
|  | Education Level 3 or 4  (Ref=≤Level 2) | 0.261 | 0.044 | 0.174 | 0.348 | 0.000 | NA |
|  | Education ≥ Level 5  (Ref=≤Level 2) | 0.490 | 0.036 | 0.419 | 0.562 | 0.000 | NA |
|  | Standing Occupation (Ref=Sitting Occupation) | 0.107 | 0.047 | 0.014 | 0.199 | 0.024 | NA |
|  | Physical Work  (Ref=Sitting Occupation) | -0.120 | 0.043 | -0.205 | -0.035 | 0.006 | NA |
|  | Heavy Manual Work  (Ref=Sitting Occupation) | -0.272 | 0.082 | -0.434 | -0.111 | 0.001 | NA |
|  | Not Working  (Ref=Sitting Occupation) | -0.259 | 0.059 | -0.375 | -0.144 | 0.000 | NA |
| **Outcome: Two-Letter Cancellation Task (Visual Search Speed + Accuracy)**  **Exposure: Device-Measured Activities (per hour)** | (Intercept) | -0.225 | 0.098 | -0.418 | -0.032 | 0.022 | NA |
|  | Total Sedentary Time | 0.014 | 0.006 | 0.001 | 0.026 | 0.033 | 0.088 |
|  | Standing Time | 0.011 | 0.008 | -0.004 | 0.026 | 0.146 | 0.278 |
|  | LPA | -0.024 | 0.020 | -0.063 | 0.016 | 0.243 | NA |
|  | MVPA | 0.052 | 0.018 | 0.015 | 0.088 | 0.005 | 0.084 |
|  | Female (Ref=Male) | 0.092 | 0.015 | 0.062 | 0.121 | 0.000 | NA |
|  | Education Level 3 or 4  (Ref=≤Level 2) | 0.057 | 0.021 | 0.017 | 0.097 | 0.006 | NA |
|  | Education ≥ Level 5  (Ref=≤Level 2) | 0.124 | 0.017 | 0.091 | 0.157 | 0.000 | NA |
|  | Standing Occupation (Ref=Sitting Occupation) | -0.007 | 0.022 | -0.050 | 0.036 | 0.744 | NA |
|  | Physical Work  (Ref=Sitting Occupation) | -0.054 | 0.020 | -0.093 | -0.015 | 0.007 | NA |
|  | Heavy Manual Work  (Ref=Sitting Occupation) | -0.072 | 0.038 | -0.147 | 0.003 | 0.058 | NA |
|  | Not Working  (Ref=Sitting Occupation) | -0.166 | 0.027 | -0.220 | -0.113 | 0.000 | NA |
| **Outcome: Global Cognition**  **Exposure: Self-Reported Sedentary Behaviors (per hour)** | (Intercept) | -0.134 | 0.060 | -0.251 | -0.016 | 0.026 | NA |
|  | Watching Television | -0.006 | 0.005 | -0.017 | 0.004 | 0.241 | 0.297 |
|  | Playing Video Games | 0.015 | 0.009 | -0.002 | 0.032 | 0.079 | 0.128 |
|  | Internet Use | 0.017 | 0.006 | 0.005 | 0.029 | 0.007 | 0.029 |
|  | Reading | 0.042 | 0.008 | 0.026 | 0.058 | 0.000 | 0.000 |
|  | MVPA | 0.054 | 0.021 | 0.014 | 0.094 | 0.008 | NA |
|  | Female (Ref=Male) | 0.060 | 0.017 | 0.027 | 0.093 | 0.000 | NA |
|  | Education Level 3 or 4  (Ref=≤Level 2) | 0.135 | 0.024 | 0.089 | 0.181 | 0.000 | NA |
|  | Education ≥ Level 5  (Ref=≤Level 2) | 0.312 | 0.020 | 0.273 | 0.351 | 0.000 | NA |
|  | Standing Occupation (Ref=Sitting Occupation) | -0.017 | 0.025 | -0.066 | 0.031 | 0.487 | NA |
|  | Physical Work  (Ref=Sitting Occupation) | -0.136 | 0.022 | -0.180 | -0.092 | 0.000 | NA |
|  | Heavy Manual Work  (Ref=Sitting Occupation) | -0.167 | 0.043 | -0.251 | -0.083 | 0.000 | NA |
|  | Not Working  (Ref=Sitting Occupation) | -0.245 | 0.031 | -0.306 | -0.185 | 0.000 | NA |
| **Outcome: 10-Word Auditory Verbal Learning Test (Immediate + Delayed Recall)**  **Exposure: Self-Reported Sedentary Behaviors (per hour)** | (Intercept) | -0.094 | 0.103 | -0.296 | 0.108 | 0.361 | NA |
|  | Watching Television | -0.013 | 0.009 | -0.032 | 0.005 | 0.154 | 0.255 |
|  | Playing Video Games | 0.003 | 0.015 | -0.026 | 0.032 | 0.861 | 0.903 |
|  | Internet Use | 0.022 | 0.011 | 0.001 | 0.043 | 0.036 | 0.127 |
|  | Reading | 0.056 | 0.014 | 0.029 | 0.084 | 0.000 | 0.001 |
|  | MVPA | 0.057 | 0.035 | -0.012 | 0.126 | 0.107 | NA |
|  | Female (Ref=Male) | 0.071 | 0.029 | 0.013 | 0.128 | 0.016 | NA |
|  | Education Level 3 or 4  (Ref=≤Level 2) | 0.161 | 0.041 | 0.082 | 0.240 | 0.000 | NA |
|  | Education ≥ Level 5  (Ref=≤Level 2) | 0.436 | 0.034 | 0.369 | 0.503 | 0.000 | NA |
|  | Standing Occupation (Ref=Sitting Occupation) | -0.089 | 0.043 | -0.173 | -0.005 | 0.037 | NA |
|  | Physical Work  (Ref=Sitting Occupation) | -0.226 | 0.038 | -0.302 | -0.151 | 0.000 | NA |
|  | Heavy Manual Work  (Ref=Sitting Occupation) | -0.224 | 0.074 | -0.369 | -0.080 | 0.002 | NA |
|  | Not Working  (Ref=Sitting Occupation) | -0.310 | 0.053 | -0.414 | -0.206 | 0.000 | NA |
| **Outcome: Category Verbal Fluency Test (Animal Naming)**  **Exposure: Self-Reported Sedentary Behaviors (per hour)** | (Intercept) | -0.180 | 0.113 | -0.401 | 0.040 | 0.109 | NA |
|  | Watching Television | -0.016 | 0.010 | -0.036 | 0.004 | 0.113 | 0.340 |
|  | Playing Video Games | 0.039 | 0.016 | 0.007 | 0.071 | 0.016 | 0.083 |
|  | Internet Use | 0.015 | 0.012 | -0.008 | 0.038 | 0.193 | 0.352 |
|  | Reading | 0.071 | 0.015 | 0.041 | 0.101 | 0.000 | 0.000 |
|  | MVPA | 0.069 | 0.039 | -0.007 | 0.144 | 0.074 | NA |
|  | Female (Ref=Male) | -0.027 | 0.032 | -0.089 | 0.036 | 0.404 | NA |
|  | Education Level 3 or 4  (Ref=≤Level 2) | 0.243 | 0.044 | 0.156 | 0.330 | 0.000 | NA |
|  | Education ≥ Level 5  (Ref=≤Level 2) | 0.450 | 0.037 | 0.378 | 0.523 | 0.000 | NA |
|  | Standing Occupation (Ref=Sitting Occupation) | 0.108 | 0.047 | 0.016 | 0.199 | 0.021 | NA |
|  | Physical Work  (Ref=Sitting Occupation) | -0.111 | 0.042 | -0.194 | -0.029 | 0.008 | NA |
|  | Heavy Manual Work  (Ref=Sitting Occupation) | -0.233 | 0.080 | -0.391 | -0.075 | 0.004 | NA |
|  | Not Working  (Ref=Sitting Occupation) | -0.263 | 0.058 | -0.377 | -0.149 | 0.000 | NA |
| **Outcome: Two-Letter Cancellation Task (Visual Search Speed + Accuracy)**  **Exposure: Self-Reported Sedentary Behaviors (per hour)** | (Intercept) | -0.150 | 0.052 | -0.253 | -0.048 | 0.004 | NA |
|  | Watching Television | 0.006 | 0.005 | -0.004 | 0.015 | 0.250 | 0.358 |
|  | Playing Video Games | 0.016 | 0.008 | 0.001 | 0.030 | 0.038 | 0.088 |
|  | Internet Use | 0.012 | 0.005 | 0.001 | 0.022 | 0.028 | 0.088 |
|  | Reading | 0.013 | 0.007 | -0.001 | 0.027 | 0.072 | 0.151 |
|  | MVPA | 0.044 | 0.018 | 0.009 | 0.079 | 0.014 | NA |
|  | Female (Ref=Male) | 0.093 | 0.015 | 0.064 | 0.122 | 0.000 | NA |
|  | Education Level 3 or 4  (Ref=≤Level 2) | 0.055 | 0.021 | 0.014 | 0.095 | 0.008 | NA |
|  | Education ≥ Level 5  (Ref=≤Level 2) | 0.119 | 0.017 | 0.085 | 0.153 | 0.000 | NA |
|  | Standing Occupation (Ref=Sitting Occupation) | -0.008 | 0.022 | -0.051 | 0.034 | 0.704 | NA |
|  | Physical Work  (Ref=Sitting Occupation) | -0.058 | 0.020 | -0.097 | -0.020 | 0.003 | NA |
|  | Heavy Manual Work  (Ref=Sitting Occupation) | -0.077 | 0.037 | -0.151 | -0.004 | 0.039 | NA |
|  | Not Working  (Ref=Sitting Occupation) | -0.172 | 0.027 | -0.225 | -0.119 | 0.000 | NA |

Abbreviations: CI = Confidence Interval; LPA = Light Physical Activity; MVPA = Moderate-to-Vigourous Physical Activity; NA = Not Applicable; p_FDR_ = p-value with Benjamini-Hochberg False Discovery Rate correction; Ref = Reference Category; SE = Standard Error

**Supplemental eTable 2.** Full multivariate linear regression parameters of all 16 distinct device-measured activity monitoring and self-reported sedentary behavior sex-stratified models.

| **Model** | **Term** | **Beta** | **SE** | **Lower 95%CI** | **Upper 95%CI** | ***p*-value** | ***p_FDR_*** |
| --- | --- | --- | --- | --- | --- | --- | --- |
| **Strata: Female**  **Outcome: Global Cognition**  **Exposure: Device-Measured Activities (per hour)** | (Intercept) | -0.122 | 0.150 | -0.416 | 0.171 | 0.415 | NA |
|  | Total Sedentary Time | 0.006 | 0.010 | -0.014 | 0.025 | 0.560 | 0.609 |
|  | Standing Time | 0.006 | 0.012 | -0.016 | 0.029 | 0.580 | 0.609 |
|  | LPA | -0.016 | 0.036 | -0.086 | 0.055 | 0.663 | NA |
|  | MVPA | 0.061 | 0.031 | 0.000 | 0.122 | 0.050 | 0.087 |
|  | Education Level 3 or 4  (Ref=≤Level 2) | 0.164 | 0.031 | 0.104 | 0.224 | 0.000 | NA |
|  | Education ≥ Level 5  (Ref=≤Level 2) | 0.391 | 0.026 | 0.340 | 0.442 | 0.000 | NA |
|  | Standing Occupation (Ref=Sitting Occupation) | 0.033 | 0.031 | -0.029 | 0.094 | 0.294 | NA |
|  | Physical Work  (Ref=Sitting Occupation) | -0.081 | 0.032 | -0.145 | -0.018 | 0.012 | NA |
|  | Heavy Manual Work  (Ref=Sitting Occupation) | -0.180 | 0.136 | -0.448 | 0.087 | 0.186 | NA |
|  | Not Working  (Ref=Sitting Occupation) | -0.175 | 0.038 | -0.249 | -0.101 | 0.000 | NA |
| **Strata: Female**  **Outcome: 10-Word Auditory Verbal Learning Test (Immediate + Delayed Recall)**  **Exposure: Device-Measured Activities (per hour)** | (Intercept) | 0.042 | 0.259 | -0.465 | 0.549 | 0.872 | NA |
|  | Total Sedentary Time | -0.004 | 0.017 | -0.038 | 0.030 | 0.824 | 0.903 |
|  | Standing Time | 0.008 | 0.020 | -0.031 | 0.048 | 0.675 | 0.788 |
|  | LPA | -0.055 | 0.062 | -0.176 | 0.066 | 0.377 | NA |
|  | MVPA | 0.056 | 0.053 | -0.049 | 0.161 | 0.298 | 0.368 |
|  | Education Level 3 or 4  (Ref=≤Level 2) | 0.224 | 0.053 | 0.120 | 0.328 | 0.000 | NA |
|  | Education ≥ Level 5  (Ref=≤Level 2) | 0.526 | 0.045 | 0.438 | 0.613 | 0.000 | NA |
|  | Standing Occupation (Ref=Sitting Occupation) | -0.036 | 0.054 | -0.142 | 0.070 | 0.509 | NA |
|  | Physical Work  (Ref=Sitting Occupation) | -0.174 | 0.056 | -0.284 | -0.065 | 0.002 | NA |
|  | Heavy Manual Work  (Ref=Sitting Occupation) | -0.376 | 0.235 | -0.837 | 0.086 | 0.111 | NA |
|  | Not Working  (Ref=Sitting Occupation) | -0.220 | 0.065 | -0.348 | -0.091 | 0.001 | NA |
| **Strata: Female**  **Outcome: Category Verbal Fluency Test (Animal Naming)**  **Exposure: Device-Measured Activities (per hour)** | (Intercept) | -0.535 | 0.273 | -1.070 | 0.001 | 0.050 | NA |
|  | Total Sedentary Time | 0.021 | 0.018 | -0.015 | 0.057 | 0.246 | 0.352 |
|  | Standing Time | 0.001 | 0.021 | -0.041 | 0.043 | 0.966 | 0.966 |
|  | LPA | 0.123 | 0.065 | -0.005 | 0.251 | 0.059 | NA |
|  | MVPA | 0.046 | 0.056 | -0.065 | 0.157 | 0.415 | 0.484 |
|  | Education Level 3 or 4  (Ref=≤Level 2) | 0.252 | 0.056 | 0.142 | 0.362 | 0.000 | NA |
|  | Education ≥ Level 5  (Ref=≤Level 2) | 0.617 | 0.047 | 0.525 | 0.710 | 0.000 | NA |
|  | Standing Occupation (Ref=Sitting Occupation) | 0.183 | 0.057 | 0.071 | 0.295 | 0.001 | NA |
|  | Physical Work  (Ref=Sitting Occupation) | -0.001 | 0.059 | -0.116 | 0.115 | 0.990 | NA |
|  | Heavy Manual Work  (Ref=Sitting Occupation) | -0.027 | 0.248 | -0.514 | 0.461 | 0.915 | NA |
|  | Not Working  (Ref=Sitting Occupation) | -0.189 | 0.069 | -0.324 | -0.054 | 0.006 | NA |
| **Strata: Female**  **Outcome: Two-Letter Cancellation Task (Visual Search Speed + Accuracy)**  **Exposure: Device-Measured Activities (per hour)** | (Intercept) | -0.080 | 0.134 | -0.341 | 0.182 | 0.552 | NA |
|  | Total Sedentary Time | 0.008 | 0.009 | -0.010 | 0.025 | 0.379 | 0.442 |
|  | Standing Time | 0.007 | 0.010 | -0.013 | 0.028 | 0.487 | 0.538 |
|  | LPA | -0.046 | 0.032 | -0.109 | 0.017 | 0.149 | NA |
|  | MVPA | 0.073 | 0.028 | 0.019 | 0.128 | 0.008 | 0.084 |
|  | Education Level 3 or 4  (Ref=≤Level 2) | 0.059 | 0.027 | 0.006 | 0.113 | 0.031 | NA |
|  | Education ≥ Level 5  (Ref=≤Level 2) | 0.143 | 0.023 | 0.098 | 0.189 | 0.000 | NA |
|  | Standing Occupation (Ref=Sitting Occupation) | 0.026 | 0.028 | -0.029 | 0.081 | 0.346 | NA |
|  | Physical Work  (Ref=Sitting Occupation) | -0.029 | 0.029 | -0.086 | 0.027 | 0.314 | NA |
|  | Heavy Manual Work  (Ref=Sitting Occupation) | -0.062 | 0.122 | -0.300 | 0.177 | 0.611 | NA |
|  | Not Working  (Ref=Sitting Occupation) | -0.123 | 0.034 | -0.190 | -0.057 | 0.000 | NA |
| **Strata: Female**  **Outcome: Global Cognition**  **Exposure: Self-Reported Sedentary Behaviors (per hour)** | (Intercept) | -0.067 | 0.077 | -0.219 | 0.084 | 0.385 | NA |
|  | Watching Television | -0.011 | 0.008 | -0.026 | 0.004 | 0.134 | 0.176 |
|  | Playing Video Games | 0.009 | 0.013 | -0.016 | 0.034 | 0.463 | 0.540 |
|  | Internet Use | 0.013 | 0.008 | -0.004 | 0.029 | 0.127 | 0.176 |
|  | Reading | 0.031 | 0.011 | 0.010 | 0.052 | 0.004 | 0.020 |
|  | MVPA | 0.051 | 0.029 | -0.007 | 0.109 | 0.082 | NA |
|  | Education Level 3 or 4  (Ref=≤Level 2) | 0.153 | 0.031 | 0.093 | 0.213 | 0.000 | NA |
|  | Education ≥ Level 5  (Ref=≤Level 2) | 0.368 | 0.026 | 0.316 | 0.420 | 0.000 | NA |
|  | Standing Occupation (Ref=Sitting Occupation) | 0.028 | 0.031 | -0.033 | 0.088 | 0.372 | NA |
|  | Physical Work  (Ref=Sitting Occupation) | -0.086 | 0.031 | -0.147 | -0.025 | 0.006 | NA |
|  | Heavy Manual Work  (Ref=Sitting Occupation) | -0.180 | 0.135 | -0.445 | 0.085 | 0.182 | NA |
|  | Not Working  (Ref=Sitting Occupation) | -0.181 | 0.037 | -0.254 | -0.108 | 0.000 | NA |
| **Strata: Female**  **Outcome: 10-Word Auditory Verbal Learning Test (Immediate + Delayed Recall)**  **Exposure: Self-Reported Sedentary Behaviors (per hour)** | (Intercept) | 0.054 | 0.134 | -0.208 | 0.316 | 0.686 | NA |
|  | Watching Television | -0.024 | 0.013 | -0.050 | 0.002 | 0.068 | 0.143 |
|  | Playing Video Games | -0.025 | 0.022 | -0.068 | 0.018 | 0.260 | 0.364 |
|  | Internet Use | 0.015 | 0.014 | -0.013 | 0.044 | 0.284 | 0.368 |
|  | Reading | 0.040 | 0.019 | 0.003 | 0.076 | 0.032 | 0.127 |
|  | MVPA | 0.029 | 0.051 | -0.070 | 0.129 | 0.564 | NA |
|  | Education Level 3 or 4  (Ref=≤Level 2) | 0.200 | 0.053 | 0.096 | 0.304 | 0.000 | NA |
|  | Education ≥ Level 5  (Ref=≤Level 2) | 0.482 | 0.046 | 0.393 | 0.572 | 0.000 | NA |
|  | Standing Occupation (Ref=Sitting Occupation) | -0.043 | 0.053 | -0.147 | 0.062 | 0.426 | NA |
|  | Physical Work  (Ref=Sitting Occupation) | -0.182 | 0.054 | -0.288 | -0.077 | 0.001 | NA |
|  | Heavy Manual Work  (Ref=Sitting Occupation) | -0.379 | 0.234 | -0.837 | 0.079 | 0.105 | NA |
|  | Not Working  (Ref=Sitting Occupation) | -0.230 | 0.064 | -0.357 | -0.104 | 0.000 | NA |
| **Strata: Female**  **Outcome: Category Verbal Fluency Test (Animal Naming)**  **Exposure: Self-Reported Sedentary Behaviors (per hour)** | (Intercept) | -0.300 | 0.141 | -0.576 | -0.023 | 0.034 | NA |
|  | Watching Television | -0.017 | 0.014 | -0.044 | 0.010 | 0.221 | 0.352 |
|  | Playing Video Games | 0.048 | 0.023 | 0.002 | 0.094 | 0.039 | 0.164 |
|  | Internet Use | 0.018 | 0.015 | -0.012 | 0.048 | 0.240 | 0.352 |
|  | Reading | 0.064 | 0.020 | 0.025 | 0.102 | 0.001 | 0.012 |
|  | MVPA | 0.069 | 0.054 | -0.036 | 0.175 | 0.198 | NA |
|  | Education Level 3 or 4  (Ref=≤Level 2) | 0.243 | 0.056 | 0.133 | 0.353 | 0.000 | NA |
|  | Education ≥ Level 5  (Ref=≤Level 2) | 0.586 | 0.048 | 0.492 | 0.680 | 0.000 | NA |
|  | Standing Occupation (Ref=Sitting Occupation) | 0.184 | 0.056 | 0.073 | 0.294 | 0.001 | NA |
|  | Physical Work  (Ref=Sitting Occupation) | 0.019 | 0.057 | -0.092 | 0.131 | 0.732 | NA |
|  | Heavy Manual Work  (Ref=Sitting Occupation) | 0.027 | 0.246 | -0.456 | 0.511 | 0.912 | NA |
|  | Not Working  (Ref=Sitting Occupation) | -0.177 | 0.068 | -0.310 | -0.044 | 0.009 | NA |
| **Strata: Female**  **Outcome: Two-Letter Cancellation Task (Visual Search Speed + Accuracy)**  **Exposure: Self-Reported Sedentary Behaviors (per hour)** | (Intercept) | -0.072 | 0.069 | -0.208 | 0.064 | 0.299 | NA |
|  | Watching Television | 0.004 | 0.007 | -0.009 | 0.017 | 0.558 | 0.558 |
|  | Playing Video Games | 0.024 | 0.011 | 0.002 | 0.047 | 0.034 | 0.088 |
|  | Internet Use | 0.007 | 0.008 | -0.007 | 0.022 | 0.320 | 0.420 |
|  | Reading | 0.006 | 0.010 | -0.013 | 0.025 | 0.521 | 0.547 |
|  | Watching Television | 0.064 | 0.026 | 0.012 | 0.116 | 0.016 | NA |
|  | Education Level 3 or 4  (Ref=≤Level 2) | 0.060 | 0.028 | 0.006 | 0.114 | 0.029 | NA |
|  | Education ≥ Level 5  (Ref=≤Level 2) | 0.144 | 0.024 | 0.098 | 0.191 | 0.000 | NA |
|  | Standing Occupation (Ref=Sitting Occupation) | 0.020 | 0.028 | -0.035 | 0.074 | 0.473 | NA |
|  | Physical Work  (Ref=Sitting Occupation) | -0.042 | 0.028 | -0.096 | 0.013 | 0.136 | NA |
|  | Heavy Manual Work  (Ref=Sitting Occupation) | -0.085 | 0.121 | -0.322 | 0.153 | 0.484 | NA |
|  | Not Working  (Ref=Sitting Occupation) | -0.133 | 0.033 | -0.198 | -0.067 | 0.000 | NA |
| **Strata: Male**  **Outcome: Global Cognition**  **Exposure: Device-Measured Activities (per hour)** | (Intercept) | -0.357 | 0.167 | -0.685 | -0.030 | 0.033 | NA |
|  | Total Sedentary Time | 0.026 | 0.011 | 0.004 | 0.047 | 0.018 | 0.062 |
|  | Standing Time | 0.027 | 0.013 | 0.001 | 0.052 | 0.039 | 0.081 |
|  | LPA | -0.019 | 0.031 | -0.079 | 0.041 | 0.541 | NA |
|  | MVPA | 0.065 | 0.029 | 0.008 | 0.123 | 0.026 | 0.070 |
|  | Education Level 3 or 4  (Ref=≤Level 2) | 0.119 | 0.037 | 0.047 | 0.192 | 0.001 | NA |
|  | Education ≥ Level 5  (Ref=≤Level 2) | 0.261 | 0.030 | 0.204 | 0.319 | 0.000 | NA |
|  | Standing Occupation (Ref=Sitting Occupation) | -0.113 | 0.043 | -0.196 | -0.029 | 0.008 | NA |
|  | Physical Work  (Ref=Sitting Occupation) | -0.213 | 0.034 | -0.279 | -0.147 | 0.000 | NA |
|  | Heavy Manual Work  (Ref=Sitting Occupation) | -0.236 | 0.049 | -0.332 | -0.139 | 0.000 | NA |
|  | Not Working  (Ref=Sitting Occupation) | -0.376 | 0.056 | -0.486 | -0.265 | 0.000 | NA |
| **Strata: Male**  **Outcome: 10-Word Auditory Verbal Learning Test (Immediate + Delayed Recall)**  **Exposure: Device-Measured Activities (per hour)** | (Intercept) | -0.553 | 0.285 | -1.112 | 0.006 | 0.053 | NA |
|  | Total Sedentary Time | 0.040 | 0.019 | 0.003 | 0.076 | 0.033 | 0.127 |
|  | Standing Time | 0.041 | 0.022 | -0.002 | 0.084 | 0.062 | 0.143 |
|  | LPA | -0.021 | 0.052 | -0.124 | 0.082 | 0.685 | NA |
|  | MVPA | 0.100 | 0.050 | 0.002 | 0.198 | 0.047 | 0.140 |
|  | Education Level 3 or 4  (Ref=≤Level 2) | 0.115 | 0.063 | -0.009 | 0.239 | 0.068 | NA |
|  | Education ≥ Level 5  (Ref=≤Level 2) | 0.413 | 0.050 | 0.314 | 0.512 | 0.000 | NA |
|  | Standing Occupation (Ref=Sitting Occupation) | -0.190 | 0.073 | -0.332 | -0.047 | 0.009 | NA |
|  | Physical Work  (Ref=Sitting Occupation) | -0.298 | 0.057 | -0.411 | -0.186 | 0.000 | NA |
|  | Heavy Manual Work  (Ref=Sitting Occupation) | -0.280 | 0.084 | -0.445 | -0.115 | 0.001 | NA |
|  | Not Working  (Ref=Sitting Occupation) | -0.476 | 0.096 | -0.664 | -0.287 | 0.000 | NA |
| **Strata: Male**  **Outcome: Category Verbal Fluency Test (Animal Naming)**  **Exposure: Device-Measured Activities (per hour)** | (Intercept) | -0.150 | 0.323 | -0.784 | 0.483 | 0.642 | NA |
|  | Total Sedentary Time | 0.011 | 0.021 | -0.030 | 0.052 | 0.607 | 0.670 |
|  | Standing Time | 0.022 | 0.025 | -0.026 | 0.071 | 0.366 | 0.481 |
|  | LPA | -0.038 | 0.059 | -0.155 | 0.078 | 0.518 | NA |
|  | MVPA | 0.069 | 0.057 | -0.042 | 0.181 | 0.223 | 0.352 |
|  | Education Level 3 or 4  (Ref=≤Level 2) | 0.261 | 0.071 | 0.121 | 0.401 | 0.000 | NA |
|  | Education ≥ Level 5  (Ref=≤Level 2) | 0.298 | 0.057 | 0.186 | 0.410 | 0.000 | NA |
|  | Standing Occupation (Ref=Sitting Occupation) | -0.047 | 0.082 | -0.209 | 0.114 | 0.566 | NA |
|  | Physical Work  (Ref=Sitting Occupation) | -0.295 | 0.065 | -0.423 | -0.168 | 0.000 | NA |
|  | Heavy Manual Work  (Ref=Sitting Occupation) | -0.410 | 0.095 | -0.597 | -0.223 | 0.000 | NA |
|  | Not Working  (Ref=Sitting Occupation) | -0.422 | 0.109 | -0.636 | -0.208 | 0.000 | NA |
| **Strata: Male**  **Outcome: Two-Letter Cancellation Task (Visual Search Speed + Accuracy)**  **Exposure: Device-Measured Activities (per hour)** | (Intercept) | -0.265 | 0.141 | -0.542 | 0.012 | 0.061 | NA |
|  | Total Sedentary Time | 0.019 | 0.009 | 0.001 | 0.037 | 0.034 | 0.088 |
|  | Standing Time | 0.014 | 0.011 | -0.007 | 0.035 | 0.189 | 0.332 |
|  | LPA | -0.006 | 0.026 | -0.057 | 0.044 | 0.802 | NA |
|  | MVPA | 0.029 | 0.025 | -0.019 | 0.078 | 0.240 | 0.358 |
|  | Education Level 3 or 4  (Ref=≤Level 2) | 0.053 | 0.031 | -0.008 | 0.114 | 0.089 | NA |
|  | Education ≥ Level 5  (Ref=≤Level 2) | 0.092 | 0.025 | 0.043 | 0.141 | 0.000 | NA |
|  | Standing Occupation (Ref=Sitting Occupation) | -0.068 | 0.036 | -0.139 | 0.002 | 0.058 | NA |
|  | Physical Work  (Ref=Sitting Occupation) | -0.087 | 0.028 | -0.142 | -0.031 | 0.002 | NA |
|  | Heavy Manual Work  (Ref=Sitting Occupation) | -0.104 | 0.042 | -0.186 | -0.022 | 0.013 | NA |
|  | Not Working  (Ref=Sitting Occupation) | -0.252 | 0.048 | -0.346 | -0.159 | 0.000 | NA |
| **Strata: Male**  **Outcome: Global Cognition**  **Exposure: Self-Reported Sedentary Behaviors (per hour)** | (Intercept) | -0.109 | 0.088 | -0.281 | 0.063 | 0.212 | NA |
|  | Watching Television | -0.002 | 0.008 | -0.017 | 0.014 | 0.827 | 0.827 |
|  | Playing Video Games | 0.020 | 0.012 | -0.003 | 0.043 | 0.087 | 0.131 |
|  | Internet Use | 0.020 | 0.009 | 0.002 | 0.038 | 0.031 | 0.072 |
|  | Reading | 0.054 | 0.013 | 0.029 | 0.079 | 0.000 | 0.000 |
|  | MVPA | 0.047 | 0.029 | -0.010 | 0.103 | 0.104 | NA |
|  | Education Level 3 or 4  (Ref=≤Level 2) | 0.107 | 0.037 | 0.035 | 0.179 | 0.004 | NA |
|  | Education ≥ Level 5  (Ref=≤Level 2) | 0.229 | 0.030 | 0.170 | 0.288 | 0.000 | NA |
|  | Standing Occupation (Ref=Sitting Occupation) | -0.105 | 0.042 | -0.188 | -0.023 | 0.013 | NA |
|  | Physical Work  (Ref=Sitting Occupation) | -0.205 | 0.033 | -0.270 | -0.141 | 0.000 | NA |
|  | Heavy Manual Work  (Ref=Sitting Occupation) | -0.215 | 0.048 | -0.309 | -0.121 | 0.000 | NA |
|  | Not Working  (Ref=Sitting Occupation) | -0.384 | 0.056 | -0.494 | -0.274 | 0.000 | NA |
| **Strata: Male**  **Outcome: 10-Word Auditory Verbal Learning Test (Immediate + Delayed Recall)**  **Exposure: Self-Reported Sedentary Behaviors (per hour)** | (Intercept) | -0.159 | 0.150 | -0.453 | 0.136 | 0.290 | NA |
|  | Watching Television | -0.002 | 0.014 | -0.028 | 0.025 | 0.903 | 0.903 |
|  | Playing Video Games | 0.028 | 0.020 | -0.012 | 0.067 | 0.168 | 0.255 |
|  | Internet Use | 0.030 | 0.016 | -0.001 | 0.060 | 0.059 | 0.143 |
|  | Reading | 0.075 | 0.022 | 0.032 | 0.118 | 0.001 | 0.006 |
|  | MVPA | 0.073 | 0.049 | -0.023 | 0.169 | 0.138 | NA |
|  | Education Level 3 or 4  (Ref=≤Level 2) | 0.099 | 0.063 | -0.025 | 0.222 | 0.118 | NA |
|  | Education ≥ Level 5  (Ref=≤Level 2) | 0.367 | 0.052 | 0.266 | 0.468 | 0.000 | NA |
|  | Standing Occupation (Ref=Sitting Occupation) | -0.177 | 0.072 | -0.318 | -0.036 | 0.014 | NA |
|  | Physical Work  (Ref=Sitting Occupation) | -0.285 | 0.056 | -0.395 | -0.175 | 0.000 | NA |
|  | Heavy Manual Work  (Ref=Sitting Occupation) | -0.248 | 0.082 | -0.409 | -0.087 | 0.003 | NA |
|  | Not Working  (Ref=Sitting Occupation) | -0.488 | 0.096 | -0.675 | -0.300 | 0.000 | NA |
| **Strata: Male**  **Outcome: Category Verbal Fluency Test (Animal Naming)**  **Exposure: Self-Reported Sedentary Behaviors (per hour)** | (Intercept) | 0.034 | 0.170 | -0.299 | 0.367 | 0.840 | NA |
|  | Watching Television | -0.021 | 0.015 | -0.051 | 0.009 | 0.178 | 0.352 |
|  | Playing Video Games | 0.026 | 0.023 | -0.019 | 0.071 | 0.251 | 0.352 |
|  | Internet Use | 0.007 | 0.018 | -0.028 | 0.041 | 0.707 | 0.743 |
|  | Reading | 0.075 | 0.025 | 0.026 | 0.123 | 0.002 | 0.017 |
|  | MVPA | 0.046 | 0.055 | -0.063 | 0.155 | 0.410 | NA |
|  | Education Level 3 or 4  (Ref=≤Level 2) | 0.239 | 0.071 | 0.099 | 0.379 | 0.001 | NA |
|  | Education ≥ Level 5  (Ref=≤Level 2) | 0.251 | 0.058 | 0.136 | 0.365 | 0.000 | NA |
|  | Standing Occupation (Ref=Sitting Occupation) | -0.043 | 0.081 | -0.203 | 0.117 | 0.596 | NA |
|  | Physical Work  (Ref=Sitting Occupation) | -0.292 | 0.064 | -0.417 | -0.167 | 0.000 | NA |
|  | Heavy Manual Work  (Ref=Sitting Occupation) | -0.387 | 0.093 | -0.569 | -0.205 | 0.000 | NA |
|  | Not Working  (Ref=Sitting Occupation) | -0.434 | 0.108 | -0.646 | -0.222 | 0.000 | NA |
| **Strata: Male**  **Outcome: Two-Letter Cancellation Task (Visual Search Speed + Accuracy)**  **Exposure: Self-Reported Sedentary Behaviors (per hour)** | (Intercept) | -0.132 | 0.074 | -0.278 | 0.014 | 0.077 | NA |
|  | Watching Television | 0.008 | 0.007 | -0.006 | 0.021 | 0.255 | 0.358 |
|  | Playing Video Games | 0.009 | 0.010 | -0.010 | 0.029 | 0.344 | 0.424 |
|  | Internet Use | 0.017 | 0.008 | 0.001 | 0.032 | 0.033 | 0.088 |
|  | Reading | 0.023 | 0.011 | 0.002 | 0.045 | 0.032 | 0.088 |
|  | MVPA | 0.021 | 0.024 | -0.027 | 0.069 | 0.394 | NA |
|  | Education Level 3 or 4  (Ref=≤Level 2) | 0.050 | 0.031 | -0.011 | 0.111 | 0.110 | NA |
|  | Education ≥ Level 5  (Ref=≤Level 2) | 0.080 | 0.026 | 0.030 | 0.130 | 0.002 | NA |
|  | Standing Occupation (Ref=Sitting Occupation) | -0.064 | 0.036 | -0.134 | 0.006 | 0.075 | NA |
|  | Physical Work  (Ref=Sitting Occupation) | -0.082 | 0.028 | -0.137 | -0.027 | 0.003 | NA |
|  | Heavy Manual Work  (Ref=Sitting Occupation) | -0.096 | 0.041 | -0.175 | -0.016 | 0.019 | NA |
|  | Not Working  (Ref=Sitting Occupation) | -0.255 | 0.047 | -0.348 | -0.162 | 0.000 | NA |

Abbreviations: CI = Confidence Interval; LPA = Light Physical Activity; MVPA = Moderate-to-Vigourous Physical Activity; NA = Not Applicable; p_FDR_ = p-value with Benjamini-Hochberg False Discovery Rate correction; Ref = Reference Category; SE = Standard Error

**Supplemental eTable 3.** Prolonged sedentary time device-measured activity monitoring sensitivity analysis models (pooled and sex-stratified).

| **Model** | **Term** | **Beta** | **SE** | **Lower 95%CI** | **Upper 95%CI** | ***p*-value** | ***p_FDR_*** |
| --- | --- | --- | --- | --- | --- | --- | --- |
| **Outcome: Global Cognition**  **Exposure: Device-Measured Activities (per hour)** | (Intercept) | -0.298 | 0.114 | -0.521 | -0.076 | 0.009 | NA |
|  | Prolonged Sedentary Time | 0.014 | 0.009 | -0.003 | 0.031 | 0.110 | 0.146 |
|  | Non-Prolonged Sedentary Time | 0.018 | 0.008 | 0.002 | 0.034 | 0.024 | 0.091 |
|  | Standing Time | 0.017 | 0.009 | 0.000 | 0.034 | 0.047 | 0.092 |
|  | LPA | -0.021 | 0.023 | -0.067 | 0.025 | 0.369 | NA |
|  | MVPA | 0.068 | 0.021 | 0.026 | 0.109 | 0.002 | 0.019 |
|  | Female (Ref=Male) | 0.070 | 0.017 | 0.036 | 0.103 | 0.000 | NA |
|  | Education Level 3 or 4  (Ref=≤Level 2) | 0.148 | 0.024 | 0.101 | 0.194 | 0.000 | NA |
|  | Education ≥ Level 5  (Ref=≤Level 2) | 0.340 | 0.019 | 0.302 | 0.378 | 0.000 | NA |
|  | Standing Occupation (Ref=Sitting Occupation) | -0.017 | 0.025 | -0.066 | 0.032 | 0.498 | NA |
|  | Physical Work  (Ref=Sitting Occupation) | -0.136 | 0.023 | -0.182 | -0.091 | 0.000 | NA |
|  | Heavy Manual Work  (Ref=Sitting Occupation) | -0.178 | 0.044 | -0.264 | -0.092 | 0.000 | NA |
|  | Not Working  (Ref=Sitting Occupation) | -0.238 | 0.031 | -0.300 | -0.177 | 0.000 | NA |
| **Outcome: 10-Word Auditory Verbal Learning Test (Immediate + Delayed Recall)**  **Exposure: Device-Measured Activities (per hour)** | (Intercept) | -0.310 | 0.195 | -0.692 | 0.072 | 0.112 | NA |
|  | Prolonged Sedentary Time | 0.013 | 0.015 | -0.016 | 0.042 | 0.389 | 0.519 |
|  | Non-Prolonged Sedentary Time | 0.021 | 0.014 | -0.006 | 0.047 | 0.134 | 0.229 |
|  | Standing Time | 0.025 | 0.015 | -0.004 | 0.054 | 0.092 | 0.220 |
|  | LPA | -0.042 | 0.040 | -0.120 | 0.037 | 0.302 | NA |
|  | MVPA | 0.080 | 0.037 | 0.008 | 0.152 | 0.029 | 0.172 |
|  | Female (Ref=Male) | 0.085 | 0.029 | 0.027 | 0.142 | 0.004 | NA |
|  | Education Level 3 or 4  (Ref=≤Level 2) | 0.182 | 0.041 | 0.102 | 0.261 | 0.000 | NA |
|  | Education ≥ Level 5  (Ref=≤Level 2) | 0.481 | 0.033 | 0.416 | 0.546 | 0.000 | NA |
|  | Standing Occupation (Ref=Sitting Occupation) | -0.090 | 0.043 | -0.174 | -0.005 | 0.038 | NA |
|  | Physical Work  (Ref=Sitting Occupation) | -0.228 | 0.040 | -0.306 | -0.150 | 0.000 | NA |
|  | Heavy Manual Work  (Ref=Sitting Occupation) | -0.238 | 0.076 | -0.386 | -0.090 | 0.002 | NA |
|  | Not Working  (Ref=Sitting Occupation) | -0.301 | 0.054 | -0.406 | -0.195 | 0.000 | NA |
| **Outcome: Category Verbal Fluency Test (Animal Naming)**  **Exposure: Device-Measured Activities (per hour)** | (Intercept) | -0.415 | 0.213 | -0.833 | 0.003 | 0.052 | NA |
|  | Prolonged Sedentary Time | 0.020 | 0.016 | -0.012 | 0.051 | 0.225 | 0.558 |
|  | Non-Prolonged Sedentary Time | 0.020 | 0.015 | -0.010 | 0.049 | 0.192 | 0.558 |
|  | Standing Time | 0.014 | 0.016 | -0.018 | 0.046 | 0.395 | 0.572 |
|  | LPA | 0.027 | 0.044 | -0.059 | 0.113 | 0.536 | NA |
|  | MVPA | 0.075 | 0.040 | -0.003 | 0.154 | 0.060 | 0.558 |
|  | Female (Ref=Male) | -0.004 | 0.032 | -0.066 | 0.059 | 0.911 | NA |
|  | Education Level 3 or 4  (Ref=≤Level 2) | 0.261 | 0.044 | 0.174 | 0.348 | 0.000 | NA |
|  | Education ≥ Level 5  (Ref=≤Level 2) | 0.490 | 0.036 | 0.419 | 0.562 | 0.000 | NA |
|  | Standing Occupation (Ref=Sitting Occupation) | 0.107 | 0.047 | 0.014 | 0.200 | 0.024 | NA |
|  | Physical Work  (Ref=Sitting Occupation) | -0.120 | 0.044 | -0.205 | -0.034 | 0.006 | NA |
|  | Heavy Manual Work  (Ref=Sitting Occupation) | -0.272 | 0.083 | -0.435 | -0.110 | 0.001 | NA |
|  | Not Working  (Ref=Sitting Occupation) | -0.259 | 0.059 | -0.375 | -0.144 | 0.000 | NA |
| **Outcome: Two-Letter Cancellation Task (Visual Search Speed + Accuracy)**  **Exposure: Device-Measured Activities (per hour)** | (Intercept) | -0.229 | 0.099 | -0.422 | -0.035 | 0.021 | NA |
|  | Prolonged Sedentary Time | 0.012 | 0.007 | -0.003 | 0.026 | 0.111 | 0.223 |
|  | Non-Prolonged Sedentary Time | 0.015 | 0.007 | 0.001 | 0.029 | 0.032 | 0.097 |
|  | Standing Time | 0.011 | 0.008 | -0.004 | 0.026 | 0.145 | 0.249 |
|  | LPA | -0.025 | 0.020 | -0.065 | 0.015 | 0.226 | NA |
|  | MVPA | 0.051 | 0.019 | 0.015 | 0.087 | 0.006 | 0.052 |
|  | Female (Ref=Male) | 0.092 | 0.015 | 0.063 | 0.121 | 0.000 | NA |
|  | Education Level 3 or 4  (Ref=≤Level 2) | 0.057 | 0.021 | 0.017 | 0.097 | 0.006 | NA |
|  | Education ≥ Level 5  (Ref=≤Level 2) | 0.125 | 0.017 | 0.092 | 0.158 | 0.000 | NA |
|  | Standing Occupation (Ref=Sitting Occupation) | -0.007 | 0.022 | -0.049 | 0.036 | 0.766 | NA |
|  | Physical Work  (Ref=Sitting Occupation) | -0.053 | 0.020 | -0.093 | -0.014 | 0.008 | NA |
|  | Heavy Manual Work  (Ref=Sitting Occupation) | -0.071 | 0.038 | -0.146 | 0.004 | 0.064 | NA |
|  | Not Working  (Ref=Sitting Occupation) | -0.165 | 0.027 | -0.219 | -0.112 | 0.000 | NA |
| **Strata: Female**  **Outcome: Global Cognition**  **Exposure: Device-Measured Activities (per hour)** | (Intercept) | -0.129 | 0.151 | -0.425 | 0.166 | 0.391 | NA |
|  | Prolonged Sedentary Time | 0.003 | 0.012 | -0.020 | 0.026 | 0.792 | 0.792 |
|  | Non-Prolonged Sedentary Time | 0.008 | 0.011 | -0.014 | 0.030 | 0.475 | 0.570 |
|  | Standing Time | 0.007 | 0.012 | -0.016 | 0.030 | 0.570 | 0.622 |
|  | LPA | -0.017 | 0.036 | -0.088 | 0.053 | 0.627 | NA |
|  | MVPA | 0.060 | 0.031 | -0.001 | 0.121 | 0.054 | 0.092 |
|  | Education Level 3 or 4  (Ref=≤Level 2) | 0.164 | 0.031 | 0.104 | 0.225 | 0.000 | NA |
|  | Education ≥ Level 5  (Ref=≤Level 2) | 0.392 | 0.026 | 0.341 | 0.442 | 0.000 | NA |
|  | Standing Occupation (Ref=Sitting Occupation) | 0.034 | 0.031 | -0.028 | 0.096 | 0.280 | NA |
|  | Physical Work  (Ref=Sitting Occupation) | -0.080 | 0.032 | -0.144 | -0.017 | 0.014 | NA |
|  | Heavy Manual Work  (Ref=Sitting Occupation) | -0.177 | 0.136 | -0.445 | 0.090 | 0.194 | NA |
|  | Not Working  (Ref=Sitting Occupation) | -0.173 | 0.038 | -0.248 | -0.099 | 0.000 | NA |
| **Strata: Female**  **Outcome: 10-Word Auditory Verbal Learning Test (Immediate + Delayed Recall)**  **Exposure: Device-Measured Activities (per hour)** | (Intercept) | 0.033 | 0.260 | -0.477 | 0.543 | 0.899 | NA |
|  | Prolonged Sedentary Time | -0.007 | 0.020 | -0.047 | 0.033 | 0.726 | 0.792 |
|  | Non-Prolonged Sedentary Time | -0.001 | 0.019 | -0.039 | 0.036 | 0.945 | 0.945 |
|  | Standing Time | 0.009 | 0.020 | -0.031 | 0.048 | 0.668 | 0.792 |
|  | LPA | -0.057 | 0.062 | -0.179 | 0.065 | 0.360 | NA |
|  | MVPA | 0.055 | 0.054 | -0.051 | 0.160 | 0.309 | 0.463 |
|  | Education Level 3 or 4  (Ref=≤Level 2) | 0.225 | 0.053 | 0.121 | 0.329 | 0.000 | NA |
|  | Education ≥ Level 5  (Ref=≤Level 2) | 0.526 | 0.045 | 0.438 | 0.614 | 0.000 | NA |
|  | Standing Occupation (Ref=Sitting Occupation) | -0.034 | 0.054 | -0.141 | 0.072 | 0.526 | NA |
|  | Physical Work  (Ref=Sitting Occupation) | -0.173 | 0.056 | -0.282 | -0.063 | 0.002 | NA |
|  | Heavy Manual Work  (Ref=Sitting Occupation) | -0.372 | 0.236 | -0.834 | 0.090 | 0.115 | NA |
|  | Not Working  (Ref=Sitting Occupation) | -0.217 | 0.066 | -0.347 | -0.088 | 0.001 | NA |
| **Strata: Female**  **Outcome: Category Verbal Fluency Test (Animal Naming)**  **Exposure: Device-Measured Activities (per hour)** | (Intercept) | -0.545 | 0.275 | -1.083 | -0.006 | 0.047 | NA |
|  | Prolonged Sedentary Time | 0.017 | 0.021 | -0.025 | 0.059 | 0.422 | 0.572 |
|  | Non-Prolonged Sedentary Time | 0.024 | 0.020 | -0.015 | 0.064 | 0.232 | 0.558 |
|  | Standing Time | 0.001 | 0.021 | -0.041 | 0.043 | 0.956 | 0.956 |
|  | LPA | 0.121 | 0.066 | -0.008 | 0.249 | 0.066 | NA |
|  | MVPA | 0.045 | 0.057 | -0.066 | 0.156 | 0.429 | 0.572 |
|  | Education Level 3 or 4  (Ref=≤Level 2) | 0.252 | 0.056 | 0.143 | 0.362 | 0.000 | NA |
|  | Education ≥ Level 5  (Ref=≤Level 2) | 0.618 | 0.047 | 0.525 | 0.711 | 0.000 | NA |
|  | Standing Occupation (Ref=Sitting Occupation) | 0.185 | 0.057 | 0.072 | 0.297 | 0.001 | NA |
|  | Physical Work  (Ref=Sitting Occupation) | 0.001 | 0.059 | -0.115 | 0.117 | 0.985 | NA |
|  | Heavy Manual Work  (Ref=Sitting Occupation) | -0.022 | 0.249 | -0.510 | 0.466 | 0.928 | NA |
|  | Not Working  (Ref=Sitting Occupation) | -0.187 | 0.069 | -0.323 | -0.050 | 0.007 | NA |
| **Strata: Female**  **Outcome: Two-Letter Cancellation Task (Visual Search Speed + Accuracy)**  **Exposure: Device-Measured Activities (per hour)** | (Intercept) | -0.084 | 0.134 | -0.347 | 0.180 | 0.534 | NA |
|  | Prolonged Sedentary Time | 0.006 | 0.010 | -0.014 | 0.027 | 0.551 | 0.551 |
|  | Non-Prolonged Sedentary Time | 0.009 | 0.010 | -0.010 | 0.028 | 0.360 | 0.432 |
|  | Standing Time | 0.007 | 0.010 | -0.013 | 0.028 | 0.481 | 0.525 |
|  | LPA | -0.047 | 0.032 | -0.110 | 0.016 | 0.143 | NA |
|  | MVPA | 0.073 | 0.028 | 0.019 | 0.127 | 0.009 | 0.052 |
|  | Education Level 3 or 4  (Ref=≤Level 2) | 0.060 | 0.027 | 0.006 | 0.113 | 0.030 | NA |
|  | Education ≥ Level 5  (Ref=≤Level 2) | 0.144 | 0.023 | 0.098 | 0.189 | 0.000 | NA |
|  | Standing Occupation (Ref=Sitting Occupation) | 0.027 | 0.028 | -0.028 | 0.082 | 0.337 | NA |
|  | Physical Work  (Ref=Sitting Occupation) | -0.028 | 0.029 | -0.085 | 0.028 | 0.329 | NA |
|  | Heavy Manual Work  (Ref=Sitting Occupation) | -0.060 | 0.122 | -0.299 | 0.179 | 0.621 | NA |
|  | Not Working  (Ref=Sitting Occupation) | -0.123 | 0.034 | -0.189 | -0.056 | 0.000 | NA |
| **Strata: Male**  **Outcome: Global Cognition**  **Exposure: Device-Measured Activities (per hour)** | (Intercept) | -0.361 | 0.167 | -0.689 | -0.033 | 0.031 | NA |
|  | Prolonged Sedentary Time | 0.023 | 0.013 | -0.002 | 0.047 | 0.073 | 0.110 |
|  | Non-Prolonged Sedentary Time | 0.028 | 0.012 | 0.005 | 0.051 | 0.017 | 0.091 |
|  | Standing Time | 0.026 | 0.013 | 0.001 | 0.052 | 0.040 | 0.092 |
|  | LPA | -0.020 | 0.031 | -0.081 | 0.040 | 0.509 | NA |
|  | MVPA | 0.064 | 0.030 | 0.006 | 0.122 | 0.030 | 0.091 |
|  | Education Level 3 or 4  (Ref=≤Level 2) | 0.119 | 0.037 | 0.047 | 0.192 | 0.001 | NA |
|  | Education ≥ Level 5  (Ref=≤Level 2) | 0.261 | 0.030 | 0.203 | 0.319 | 0.000 | NA |
|  | Standing Occupation (Ref=Sitting Occupation) | -0.112 | 0.043 | -0.195 | -0.028 | 0.009 | NA |
|  | Physical Work  (Ref=Sitting Occupation) | -0.212 | 0.034 | -0.278 | -0.146 | 0.000 | NA |
|  | Heavy Manual Work  (Ref=Sitting Occupation) | -0.234 | 0.049 | -0.331 | -0.137 | 0.000 | NA |
|  | Not Working  (Ref=Sitting Occupation) | -0.373 | 0.057 | -0.484 | -0.262 | 0.000 | NA |
| **Strata: Male**  **Outcome: 10-Word Auditory Verbal Learning Test (Immediate + Delayed Recall)**  **Exposure: Device-Measured Activities (per hour)** | (Intercept) | -0.560 | 0.286 | -1.120 | -0.000 | 0.050 | NA |
|  | Prolonged Sedentary Time | 0.033 | 0.021 | -0.009 | 0.075 | 0.122 | 0.229 |
|  | Non-Prolonged Sedentary Time | 0.044 | 0.020 | 0.005 | 0.082 | 0.028 | 0.172 |
|  | Standing Time | 0.041 | 0.022 | -0.002 | 0.084 | 0.064 | 0.193 |
|  | LPA | -0.024 | 0.053 | -0.128 | 0.079 | 0.644 | NA |
|  | MVPA | 0.097 | 0.050 | -0.002 | 0.196 | 0.055 | 0.193 |
|  | Education Level 3 or 4  (Ref=≤Level 2) | 0.115 | 0.063 | -0.009 | 0.238 | 0.069 | NA |
|  | Education ≥ Level 5  (Ref=≤Level 2) | 0.412 | 0.050 | 0.313 | 0.511 | 0.000 | NA |
|  | Standing Occupation (Ref=Sitting Occupation) | -0.188 | 0.073 | -0.331 | -0.046 | 0.010 | NA |
|  | Physical Work  (Ref=Sitting Occupation) | -0.297 | 0.057 | -0.409 | -0.184 | 0.000 | NA |
|  | Heavy Manual Work  (Ref=Sitting Occupation) | -0.277 | 0.084 | -0.442 | -0.111 | 0.001 | NA |
|  | Not Working  (Ref=Sitting Occupation) | -0.471 | 0.097 | -0.660 | -0.282 | 0.000 | NA |
| **Strata: Male**  **Outcome: Category Verbal Fluency Test (Animal Naming)**  **Exposure: Device-Measured Activities (per hour)** | (Intercept) | -0.149 | 0.323 | -0.783 | 0.486 | 0.646 | NA |
|  | Prolonged Sedentary Time | 0.012 | 0.024 | -0.035 | 0.060 | 0.613 | 0.722 |
|  | Non-Prolonged Sedentary Time | 0.010 | 0.022 | -0.034 | 0.054 | 0.661 | 0.722 |
|  | Standing Time | 0.023 | 0.025 | -0.026 | 0.071 | 0.365 | 0.572 |
|  | LPA | -0.038 | 0.060 | -0.155 | 0.079 | 0.528 | NA |
|  | MVPA | 0.070 | 0.057 | -0.042 | 0.182 | 0.221 | 0.558 |
|  | Education Level 3 or 4  (Ref=≤Level 2) | 0.261 | 0.071 | 0.121 | 0.401 | 0.000 | NA |
|  | Education ≥ Level 5  (Ref=≤Level 2) | 0.298 | 0.057 | 0.186 | 0.410 | 0.000 | NA |
|  | Standing Occupation (Ref=Sitting Occupation) | -0.047 | 0.082 | -0.209 | 0.114 | 0.564 | NA |
|  | Physical Work  (Ref=Sitting Occupation) | -0.296 | 0.065 | -0.423 | -0.168 | 0.000 | NA |
|  | Heavy Manual Work  (Ref=Sitting Occupation) | -0.410 | 0.096 | -0.598 | -0.223 | 0.000 | NA |
|  | Not Working  (Ref=Sitting Occupation) | -0.423 | 0.109 | -0.638 | -0.209 | 0.000 | NA |
| **Strata: Male**  **Outcome: Two-Letter Cancellation Task (Visual Search Speed + Accuracy)**  **Exposure: Device-Measured Activities (per hour)** | (Intercept) | -0.268 | 0.141 | -0.545 | 0.009 | 0.058 | NA |
|  | Prolonged Sedentary Time | 0.017 | 0.011 | -0.004 | 0.038 | 0.111 | 0.223 |
|  | Non-Prolonged Sedentary Time | 0.021 | 0.010 | 0.002 | 0.040 | 0.032 | 0.097 |
|  | Standing Time | 0.014 | 0.011 | -0.007 | 0.035 | 0.194 | 0.291 |
|  | LPA | -0.008 | 0.026 | -0.059 | 0.043 | 0.768 | NA |
|  | MVPA | 0.028 | 0.025 | -0.021 | 0.077 | 0.261 | 0.348 |
|  | Education Level 3 or 4  (Ref=≤Level 2) | 0.053 | 0.031 | -0.008 | 0.114 | 0.090 | NA |
|  | Education ≥ Level 5  (Ref=≤Level 2) | 0.091 | 0.025 | 0.043 | 0.140 | 0.000 | NA |
|  | Standing Occupation (Ref=Sitting Occupation) | -0.068 | 0.036 | -0.138 | 0.003 | 0.060 | NA |
|  | Physical Work  (Ref=Sitting Occupation) | -0.086 | 0.028 | -0.142 | -0.030 | 0.002 | NA |
|  | Heavy Manual Work  (Ref=Sitting Occupation) | -0.103 | 0.042 | -0.185 | -0.021 | 0.014 | NA |
|  | Not Working  (Ref=Sitting Occupation) | -0.251 | 0.048 | -0.344 | -0.157 | 0.000 | NA |

Abbreviations: CI = Confidence Interval; LPA = Light Physical Activity; MVPA = Moderate-to-Vigourous Physical Activity; NA = Not Applicable; p_FDR_ = p-value with Benjamini-Hochberg False Discovery Rate correction; Ref = Reference Category; SE = Standard Error

**Supplemental eTable 4.** Unadjusted device-measured activities models.

| **Model** | **Term** | **Beta** | **SE** | **Lower 95%CI** | **Upper 95%CI** | ***p*-value** |
| --- | --- | --- | --- | --- | --- | --- |
| **Outcome: Global Cognition**  **Exposure: Device-Measured Activities (per hour)** | (Intercept) | -0.162 | 0.117 | -0.390 | 0.067 | 0.166 |
|  | Total Sedentary Time | 0.020 | 0.008 | 0.005 | 0.035 | 0.010 |
|  | Standing Time | 0.013 | 0.009 | -0.005 | 0.031 | 0.158 |
|  | LPA | -0.109 | 0.023 | -0.155 | -0.063 | 0.000 |
|  | MVPA | 0.137 | 0.022 | 0.093 | 0.181 | 0.000 |
| **Outcome: 10-Word Auditory Verbal Learning Test (Immediate + Delayed Recall)**  **Exposure: Device-Measured Activities (per hour)** | (Intercept) | -0.146 | 0.196 | -0.530 | 0.237 | 0.455 |
|  | Total Sedentary Time | 0.025 | 0.013 | -0.001 | 0.050 | 0.055 |
|  | Standing Time | 0.016 | 0.015 | -0.014 | 0.046 | 0.302 |
|  | LPA | -0.165 | 0.039 | -0.242 | -0.088 | 0.000 |
|  | MVPA | 0.174 | 0.038 | 0.100 | 0.248 | 0.000 |
| **Outcome: Category Verbal Fluency Test (Animal Naming)**  **Exposure: Device-Measured Activities (per hour)** | (Intercept) | -0.322 | 0.213 | -0.739 | 0.095 | 0.130 |
|  | Total Sedentary Time | 0.028 | 0.014 | 0.000 | 0.055 | 0.047 |
|  | Standing Time | 0.012 | 0.017 | -0.021 | 0.044 | 0.481 |
|  | LPA | -0.061 | 0.043 | -0.144 | 0.023 | 0.155 |
|  | MVPA | 0.170 | 0.041 | 0.090 | 0.250 | 0.000 |
| **Outcome: Two-Letter Cancellation Task (Visual Search Speed + Accuracy)**  **Exposure: Device-Measured Activities (per hour)** | (Intercept) | -0.097 | 0.097 | -0.287 | 0.093 | 0.317 |
|  | Total Sedentary Time | 0.011 | 0.006 | -0.002 | 0.023 | 0.094 |
|  | Standing Time | 0.011 | 0.008 | -0.004 | 0.025 | 0.166 |
|  | LPA | -0.077 | 0.019 | -0.115 | -0.039 | 0.000 |
|  | MVPA | 0.083 | 0.019 | 0.047 | 0.120 | 0.000 |
| **Outcome: Global Cognition**  **Exposure: Self-Reported Sedentary Behaviors (per hour)** | (Intercept) | -0.090 | 0.042 | -0.173 | -0.007 | 0.033 |
|  | Watching Television | -0.006 | 0.005 | -0.016 | 0.004 | 0.223 |
|  | Playing Video Games | 0.000 | 0.009 | -0.017 | 0.017 | 0.975 |
|  | Internet Use | 0.034 | 0.006 | 0.023 | 0.046 | 0.000 |
|  | Reading | 0.081 | 0.008 | 0.065 | 0.097 | 0.000 |
| **Outcome: 10-Word Auditory Verbal Learning Test (Immediate + Delayed Recall)**  **Exposure: Self-Reported Sedentary Behaviors (per hour)** | (Intercept) | -0.132 | 0.071 | -0.272 | 0.007 | 0.063 |
|  | Watching Television | -0.007 | 0.009 | -0.024 | 0.010 | 0.433 |
|  | Playing Video Games | -0.012 | 0.015 | -0.041 | 0.017 | 0.407 |
|  | Internet Use | 0.053 | 0.010 | 0.034 | 0.073 | 0.000 |
|  | Reading | 0.115 | 0.014 | 0.088 | 0.142 | 0.000 |
| **Outcome: Category Verbal Fluency Test (Animal Naming)**  **Exposure: Self-Reported Sedentary Behaviors (per hour)** | (Intercept) | -0.096 | 0.077 | -0.247 | 0.056 | 0.215 |
|  | Watching Television | -0.019 | 0.009 | -0.038 | -0.001 | 0.041 |
|  | Playing Video Games | 0.020 | 0.016 | -0.011 | 0.052 | 0.199 |
|  | Internet Use | 0.037 | 0.011 | 0.016 | 0.058 | 0.001 |
|  | Reading | 0.115 | 0.015 | 0.086 | 0.144 | 0.000 |
| **Outcome: Two-Letter Cancellation Task (Visual Search Speed + Accuracy)**  **Exposure: Self-Reported Sedentary Behaviors (per hour)** | (Intercept) | -0.045 | 0.036 | -0.115 | 0.025 | 0.204 |
|  | Watching Television | 0.001 | 0.004 | -0.008 | 0.009 | 0.864 |
|  | Playing Video Games | 0.003 | 0.007 | -0.012 | 0.017 | 0.721 |
|  | Internet Use | 0.014 | 0.005 | 0.004 | 0.024 | 0.004 |
|  | Reading | 0.029 | 0.007 | 0.015 | 0.042 | 0.000 |

**Supplemental eTable 5.** Unadjusted self-reported sedentary behavior models.

| **Model** | **Term** | **Beta** | **SE** | **Lower 95%CI** | **Upper 95%CI** | ***p*-value** |
| --- | --- | --- | --- | --- | --- | --- |
| **Strata: Female**  **Outcome: Global Cognition**  **Exposure: Device-Measured Activities (per hour)** | (Intercept) | -0.071 | 0.159 | -0.383 | 0.241 | 0.655 |
|  | Total Sedentary Time | 0.012 | 0.011 | -0.009 | 0.033 | 0.248 |
|  | Standing Time | 0.004 | 0.012 | -0.020 | 0.028 | 0.738 |
|  | LPA | -0.062 | 0.037 | -0.135 | 0.010 | 0.089 |
|  | MVPA | 0.142 | 0.033 | 0.078 | 0.206 | 0.000 |
| **Strata: Female**  **Outcome: 10-Word Auditory Verbal Learning Test (Immediate + Delayed Recall)**  **Exposure: Device-Measured Activities (per hour)** | (Intercept) | 0.099 | 0.268 | -0.427 | 0.624 | 0.713 |
|  | Total Sedentary Time | 0.007 | 0.018 | -0.028 | 0.042 | 0.678 |
|  | Standing Time | 0.002 | 0.021 | -0.039 | 0.042 | 0.940 |
|  | LPA | -0.135 | 0.062 | -0.256 | -0.013 | 0.030 |
|  | MVPA | 0.163 | 0.055 | 0.055 | 0.270 | 0.003 |
| **Strata: Female**  **Outcome: Category Verbal Fluency Test (Animal Naming)**  **Exposure: Device-Measured Activities (per hour)** | (Intercept) | -0.408 | 0.285 | -0.966 | 0.150 | 0.152 |
|  | Total Sedentary Time | 0.026 | 0.019 | -0.011 | 0.063 | 0.170 |
|  | Standing Time | 0.001 | 0.022 | -0.042 | 0.045 | 0.953 |
|  | LPA | 0.092 | 0.066 | -0.037 | 0.221 | 0.162 |
|  | MVPA | 0.165 | 0.058 | 0.051 | 0.280 | 0.005 |
| **Strata: Female**  **Outcome: Two-Letter Cancellation Task (Visual Search Speed + Accuracy)**  **Exposure: Device-Measured Activities (per hour)** | (Intercept) | -0.072 | 0.135 | -0.337 | 0.192 | 0.591 |
|  | Total Sedentary Time | 0.010 | 0.009 | -0.007 | 0.028 | 0.258 |
|  | Standing Time | 0.008 | 0.010 | -0.012 | 0.029 | 0.438 |
|  | LPA | -0.068 | 0.031 | -0.129 | -0.007 | 0.030 |
|  | MVPA | 0.109 | 0.028 | 0.055 | 0.163 | 0.000 |
| **Strata: Female**  **Outcome: Global Cognition**  **Exposure: Self-Reported Sedentary Behaviors (per hour)** | (Intercept) | 0.037 | 0.057 | -0.075 | 0.150 | 0.518 |
|  | Watching Television | -0.014 | 0.007 | -0.028 | 0.000 | 0.054 |
|  | Playing Video Games | -0.010 | 0.013 | -0.036 | 0.016 | 0.451 |
|  | Internet Use | 0.024 | 0.008 | 0.007 | 0.040 | 0.004 |
|  | Reading | 0.059 | 0.011 | 0.038 | 0.080 | 0.000 |
| **Strata: Female**  **Outcome: 10-Word Auditory Verbal Learning Test (Immediate + Delayed Recall)**  **Exposure: Self-Reported Sedentary Behaviors (per hour)** | (Intercept) | 0.039 | 0.097 | -0.151 | 0.229 | 0.687 |
|  | Watching Television | -0.017 | 0.012 | -0.041 | 0.007 | 0.171 |
|  | Playing Video Games | -0.042 | 0.022 | -0.085 | 0.001 | 0.058 |
|  | Internet Use | 0.039 | 0.014 | 0.012 | 0.067 | 0.005 |
|  | Reading | 0.086 | 0.018 | 0.051 | 0.122 | 0.000 |
| **Strata: Female**  **Outcome: Category Verbal Fluency Test (Animal Naming)**  **Exposure: Self-Reported Sedentary Behaviors (per hour)** | (Intercept) | 0.044 | 0.103 | -0.158 | 0.245 | 0.672 |
|  | Watching Television | -0.035 | 0.013 | -0.060 | -0.009 | 0.008 |
|  | Playing Video Games | 0.012 | 0.023 | -0.034 | 0.058 | 0.612 |
|  | Internet Use | 0.022 | 0.015 | -0.007 | 0.051 | 0.131 |
|  | Reading | 0.093 | 0.019 | 0.055 | 0.130 | 0.000 |
| **Strata: Female**  **Outcome: Two-Letter Cancellation Task (Visual Search Speed + Accuracy)**  **Exposure: Self-Reported Sedentary Behaviors (per hour)** | (Intercept) | 0.032 | 0.049 | -0.065 | 0.129 | 0.516 |
|  | Watching Television | -0.001 | 0.006 | -0.013 | 0.011 | 0.890 |
|  | Playing Video Games | 0.011 | 0.011 | -0.011 | 0.033 | 0.315 |
|  | Internet Use | 0.009 | 0.007 | -0.005 | 0.023 | 0.224 |
|  | Reading | 0.014 | 0.009 | -0.004 | 0.032 | 0.123 |
| **Strata: Male**  **Outcome: Global Cognition**  **Exposure: Device-Measured Activities (per hour)** | (Intercept) | -0.482 | 0.175 | -0.825 | -0.139 | 0.006 |
|  | Total Sedentary Time | 0.043 | 0.011 | 0.021 | 0.065 | 0.000 |
|  | Standing Time | 0.024 | 0.014 | -0.003 | 0.050 | 0.081 |
|  | LPA | -0.095 | 0.031 | -0.157 | -0.034 | 0.002 |
|  | MVPA | 0.128 | 0.031 | 0.067 | 0.188 | 0.000 |
| **Strata: Male**  **Outcome: 10-Word Auditory Verbal Learning Test (Immediate + Delayed Recall)**  **Exposure: Device-Measured Activities (per hour)** | (Intercept) | -0.693 | 0.294 | -1.269 | -0.117 | 0.018 |
|  | Total Sedentary Time | 0.062 | 0.019 | 0.025 | 0.100 | 0.001 |
|  | Standing Time | 0.034 | 0.023 | -0.011 | 0.078 | 0.139 |
|  | LPA | -0.125 | 0.053 | -0.229 | -0.022 | 0.018 |
|  | MVPA | 0.182 | 0.052 | 0.081 | 0.283 | 0.000 |
| **Strata: Male**  **Outcome: Category Verbal Fluency Test (Animal Naming)**  **Exposure: Device-Measured Activities (per hour)** | (Intercept) | -0.322 | 0.327 | -0.964 | 0.320 | 0.325 |
|  | Total Sedentary Time | 0.035 | 0.021 | -0.007 | 0.077 | 0.101 |
|  | Standing Time | 0.020 | 0.025 | -0.030 | 0.069 | 0.436 |
|  | LPA | -0.151 | 0.059 | -0.267 | -0.036 | 0.010 |
|  | MVPA | 0.154 | 0.058 | 0.041 | 0.267 | 0.008 |
| **Strata: Male**  **Outcome: Two-Letter Cancellation Task (Visual Search Speed + Accuracy)**  **Exposure: Device-Measured Activities (per hour)** | (Intercept) | -0.352 | 0.141 | -0.629 | -0.075 | 0.013 |
|  | Total Sedentary Time | 0.027 | 0.009 | 0.009 | 0.045 | 0.003 |
|  | Standing Time | 0.016 | 0.011 | -0.006 | 0.037 | 0.156 |
|  | LPA | -0.038 | 0.025 | -0.087 | 0.012 | 0.140 |
|  | MVPA | 0.060 | 0.025 | 0.011 | 0.109 | 0.016 |
| **Strata: Male**  **Outcome: Global Cognition**  **Exposure: Self-Reported Sedentary Behaviors (per hour)** | (Intercept) | -0.288 | 0.063 | -0.412 | -0.163 | 0.000 |
|  | Watching Television | 0.009 | 0.008 | -0.006 | 0.023 | 0.249 |
|  | Playing Video Games | 0.018 | 0.012 | -0.005 | 0.041 | 0.125 |
|  | Internet Use | 0.053 | 0.008 | 0.036 | 0.069 | 0.000 |
|  | Reading | 0.103 | 0.012 | 0.079 | 0.128 | 0.000 |
| **Strata: Male**  **Outcome: 10-Word Auditory Verbal Learning Test (Immediate + Delayed Recall)**  **Exposure: Self-Reported Sedentary Behaviors (per hour)** | (Intercept) | -0.390 | 0.107 | -0.599 | -0.180 | 0.000 |
|  | Watching Television | 0.012 | 0.013 | -0.013 | 0.037 | 0.338 |
|  | Playing Video Games | 0.023 | 0.020 | -0.016 | 0.062 | 0.239 |
|  | Internet Use | 0.076 | 0.014 | 0.049 | 0.104 | 0.000 |
|  | Reading | 0.145 | 0.021 | 0.104 | 0.186 | 0.000 |
| **Strata: Male**  **Outcome: Category Verbal Fluency Test (Animal Naming)**  **Exposure: Self-Reported Sedentary Behaviors (per hour)** | (Intercept) | -0.254 | 0.119 | -0.488 | -0.020 | 0.033 |
|  | Watching Television | -0.003 | 0.014 | -0.031 | 0.025 | 0.846 |
|  | Playing Video Games | 0.030 | 0.022 | -0.014 | 0.073 | 0.183 |
|  | Internet Use | 0.053 | 0.016 | 0.021 | 0.084 | 0.001 |
|  | Reading | 0.144 | 0.023 | 0.098 | 0.190 | 0.000 |
| **Strata: Male**  **Outcome: Two-Letter Cancellation Task (Visual Search Speed + Accuracy)**  **Exposure: Self-Reported Sedentary Behaviors (per hour)** | (Intercept) | -0.202 | 0.052 | -0.304 | -0.100 | 0.000 |
|  | Watching Television | 0.011 | 0.006 | -0.001 | 0.023 | 0.076 |
|  | Playing Video Games | 0.007 | 0.010 | -0.012 | 0.026 | 0.465 |
|  | Internet Use | 0.029 | 0.007 | 0.015 | 0.042 | 0.000 |
|  | Reading | 0.042 | 0.010 | 0.022 | 0.062 | 0.000 |

Abbreviations: CI = Confidence Interval; LPA = Light Physical Activity; MVPA = Moderate-to-Vigourous Physical Activity; SE = Standard Error
